# Metabolomics analyses reveal the crucial role of ERK in regulating metabolic pathways associated with the proliferation of human cutaneous T‐cell lymphoma cells treated with Glabridin

**DOI:** 10.1111/cpr.13701

**Published:** 2024-06-30

**Authors:** Abdul Q. Khan, Maha Victor Agha, Fareed Ahmad, Rasheeda Anver, Khalid Sultan A. M. Sheikhan, Jericha Mateo, Majid Alam, Joerg Buddenkotte, Shahab Uddin, Martin Steinhoff

**Affiliations:** ^1^ Translational Research Institute Academic Health System, Hamad Medical Corporation Doha Qatar; ^2^ Dermatology Institute, Academic Health System Hamad Medical Corporation Doha Qatar; ^3^ Department of Dermatology and Venereology Rumailah Hospital, Hamad Medical Corporation Doha Qatar; ^4^ Laboratory Animal Research Center Qatar University Doha Qatar; ^5^ Department of Medicine Weill Cornell Medicine Qatar, Qatar Foundation‐Education City Doha Qatar; ^6^ Department of Medicine Weill Cornell Medicine New York New York USA; ^7^ College of Medicine Qatar University Doha Qatar

## Abstract

Cutaneous T‐cell lymphomas (CTC) are a heterogeneous group of T‐cell lymphoproliferative malignancies of the skin with limited treatment options, increased resistance and remission. Metabolic reprogramming is vital in orchestrating the uncontrolled growth and proliferation of cancer cells. Importantly, deregulated signalling plays a significant role in metabolic reprogramming. Considering the crucial role of metabolic reprogramming in cancer‐cell growth and proliferation, target identification and the development of novel and multi‐targeting agents are imperative. The present study explores the underlying mechanisms and metabolic signalling pathways associated with Glabridin mediated anti‐cancer actions in CTCL. Our results show that Glabridin significantly inhibits the growth of CTCL cells through induction of programmed cell death (PCD) such as apoptosis, autophagy and necrosis. Interestingly, results further show that Glabridin induces PCD in CTCL cells by targeting MAPK signalling pathways, particularly the activation of ERK. Further, Glabridin also sensitized CTCL cells to the anti‐cancer drug, bortezomib. Importantly, LC–MS‐based metabolomics analyses further showed that Glabridin targeted multiple metabolites and metabolic pathways intricately involved in cancer cell growth and proliferation in an ERK‐dependent fashion. Overall, our findings revealed that Glabridin induces PCD and attenuates the expression of regulatory proteins and metabolites involved in orchestrating the uncontrolled proliferation of CTCL cells through ERK activation. Therefore, Glabridin possesses important features of an ideal anti‐cancer agent.

## Graphical abstract

The present study shows that Glabridin induces programmed cell death, metabolic regulation and spheroid inhibition through ERK activation and sensitizes CTCL cells to bortezomib.

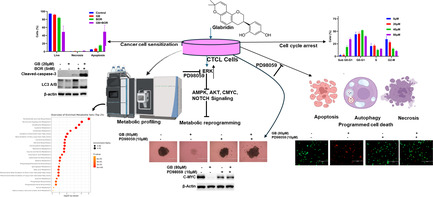

## 1 INTRODUCTION

Cancer is a complex and heterogeneous disease with exponentially increasing incidence, deaths and morbidity, posing a huge socio‐economic burden to patients and their relatives. In this line, skin cancer is a major health problem and is increasing at an alarming rate. Basal cell carcinoma and squamous cell carcinoma are the two major types of skin cancer. Cutaneous T‐cell lymphomas (CTCL), a heterogeneous group of T‐cell lymphoproliferative diseases or non‐Hodgkin's lymphomas, primarily involves the malignant T lymphocyte proliferation mainly in the skin as patches, tumours, erythrodermas and plaques.^1^ Despite its low occurrence, the death ratio is high due to limited therapeutic options and poor understanding of the CTCL disease pathogenesis. Moreover, the available treatment options are mostly symptomatic and hence require additional thorough investigation at molecular and cellular levels.

Deregulated metabolite signalling is an important contributor to the genesis of different human diseases, including cancer. Metabolic deregulation not only provides energy but also reprograms other signalling mechanisms related to promotion and progression, stemness, immunomodulation and microenvironment in cancer cells.^2^, ^3^ Indeed, like other cancer types, metabolic alterations of key metabolic enzymes through epigenetic and genetic changes are crucial in T‐cell lymphoma pathogenesis.^4^, ^5^, ^6^, ^7^ Deregulation of cell signalling due to genetic and epigenetic changes affects several pathways resulting in metabolic reprogramming and thus enabling the cancer cells to sustain growth and metastasis.^8^, ^9^, ^10^ In this line, the role of mitogen‐activated protein kinase (MAPK) signalling is crucial in developmental and physiological mechanisms and recent updates also indicate that abnormal expression of MAPK signalling is often associated with cancer pathogenesis and poor clinical outcomes including drug resistance and disease relapse.^11^, ^12^ The present study explores the status of MAPK signalling in CTCL cell lines under different experimental conditions.

Metabolic reprogramming plays an integral role in the continuous growth and proliferation of cancer cells and in orchestrating the cancer hallmarks including the stemness via modulating the various metabolic pathways including energy metabolism, amino acid metabolism, fatty acid oxidation, redox metabolism and nucleic acid.^13^, ^14^, ^15^, ^16^ Interestingly, metabolic reprogramming of immune cells is another important mechanism for cancer cell growth and progression.^17^, ^18^ Indeed, various signalling pathways have been shown to regulate the metabolic reprogramming in cancer cells, including the MAPK and AKT pathways.^10^, ^19^, ^20^, ^21^ In line, extracellular signal‐regulated kinase (ERK) signalling plays an important role in the regulation of metabolic reprogramming in cancer cells.^10^, ^22^, ^23^

Moreover, another important aspect of this investigation is to explore the development of novel and effective treatment options, as the current therapeutic measures of CTCL are not adequate and are associated with limitations. Indeed, available data advocate the use of naturally derived products/small molecule inhibitors for the treatment of different human diseases, including cancer. Glabridin, a major bioactive prenylated isoflavone present in the root of licorice (*Glycyrrhiza glabra*), possesses a range of pharmacological features (e.g., anti‐oxidant, anti‐inflammatory, anti‐microbial, anti‐obesity, anti‐tumour, metabolism of blood lipid and glucose metabolism, skin whitening etc.) and has shown promising therapeutic outcomes for various human diseases, including cancer and diabetes.^24^ Glabridin has been shown to modulate the action of different drugs due its effect of on the metabolizing enzymes like cytochromes P450 enzymes.^25^, ^26^ Glabridin also acts as oestrogen receptor agonist and sensitize the human cancer cells to anti‐cancer drugs tamoxifen and paclitaxel.^25^ Moreover, Glabridin suppress atopic dermatitis and photoaging through inhibition of inflammatory mediators via targeting inflammation associated regulatory proteins.^27^, ^28^ Mechanistically, Glabridin exert its pharmacological actions via targeting different signalling pathways, including MAPK, AKT and NF‐kB.^24^, ^29^ Importantly, the multi‐targeting potential of Glabridin supports the therapeutic potential of this compound.^24^

Using both mechanistic and metabolomics approach, we explored the ERK‐dependent regulation of metabolic pathways associated with the programmed cell death in CTCL cells treated with Glabridin.

## 2 MATERIALS AND METHODS

### 2.1 Chemicals and reagents

Cell counting kit‐8 (CCK‐8), dimethyl sulfoxide (DMSO), 3‐methyl adenine (3MA) and other important high‐grade reagents were procured from Sigma Aldrich (St. Louis, Missouri, USA). Glabridin, PD98059 and VAD (Z‐VAD‐FMK [carbobenzoxy‐valyl‐alanyl‐aspartyl‐[O‐methyl]‐fluoromethylketone]) were obtained from Selleck Chemicals (14408 W Sylvanfield Drive, Houston, TX 77014, USA). Antibodies such as caspase‐3, cleaved caspase‐3, cleaved caspase‐8, phospho‐histone H2A.X (p‐H2AX), HMGB1, P‐AKT, AKT, NOTCH, P‐AMPK, AMPK, PP38, P38, P‐ERK, ERK, P‐c‐jun N‐terminal kinase (JNK), JNK, poly‐ADP ribose polymerase (PARP), cleaved PARP, microtubule‐associated protein 1A/1B‐light chain 3 (LC3A/B), HSP60, glyceraldehyde 3‐phosphate dehydrogenase (GAPDH), β‐actin and so forth, were procured from the Cell Signalling Technology, Inc. (3 Trask Lane Danvers, MA 01923) and the Santa Cruz Biotechnology, Inc. (Finnell Street Dallas, Texas, USA) (Supplementary Table S4). Bortezomib was purchased from Cell Signalling Technology, Inc., USA. Western blotting reagents, including the laemmli sample buffer 1X, resolving and stacking gel solutions and clarity western enhanced chemiluminescence (ECL) and so forth, were obtained from the BIO‐RAD (Hercules, California, USA).

### 2.2 Chemical structure of Glabridin

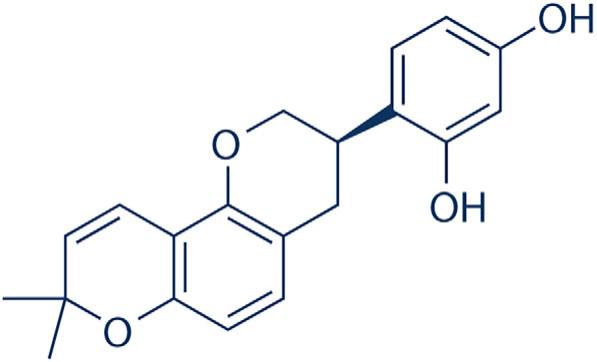

Purity = 99.87%.

### 2.3 Cell culture

Human cutaneous T‐cell lymphoma cell lines, HH and H9 were acquired from the American Type Culture Collection (ATCC), 10801 University Boulevard Manassas, VA 20110, USA. Cells were cultured in Roswell Park Memorial Institute (RPMI) 1640 Medium supplemented with 10% (v/v) fetal bovine serum (FBS), 100 U/mL penicillin and 100 U/mL streptomycin at 37°C in a humidified atmosphere containing 5% CO_2_. Treatments were done in a 5% RPMI medium.

### 2.4 Cell counting kit‐8 (CCK‐8) assay

The anti‐proliferative/cytotoxic action of Glabridin, bortezomib and or pharmacological inhibitors in CTCL cells was done using the Cell Counting Kit‐8 (CCK‐8) assay as described earlier.^30^

### 2.5 Annexin V staining

CTCL cells were treated with Glabridin alone and or in combination with bortezomib or the ERK inhibitor. After 24 h of the treatment, cells were harvested and washed with phosphate‐buffered saline (PBS). To measure the frequency of apoptotic cells, roughly 500,000 cells were stained with fluorescein‐conjugated annexin V antibody and propidium iodide for 30 min at room temperature in an annexin binding buffer. Around 300,000 stained cells were acquired by flow cytometry (BD LSRFortessa analyser, BD Biosciences). To find out the frequencies of apoptotic and necrotic cells, FCS3 files of the recorded data were analysed using FlowJo (version10.7.1) software.

### 2.6 Measurement of mitochondrial membrane potential

HH and H9 cells were treated with Glabridin alone and or in combination. After 24 h of the treatment, cells were harvested, washed and finally stained (500,000 cells) with a JC1 stain kit for 30 min at room temperature as per the manufacturer's instruction (Thermofisher) and then analysed using flow cytometry (BD LSR Fortessa analyser, BD Biosciences, USA).

### 2.7 Cell cycle analysis

HH and H9 cells were treated with Glabridin for 24 h. Thereafter, cells were harvested and fixed using 100% ethanol overnight at 4°C. The next day, cells were washed using HBSS and resuspended in 250 μL of PI/RNase solution (BD Biosciences) for 15 min at room temperature. After incubation, cells were washed and resuspended in 300 μL of HBSS. Cells were acquired on a flow cytometer BD LSRFortessa cell analyser and analysed as mentioned above.

### 2.8 Live/dead assay

CTCL cells were treated with Glabridin alone and or in combination with ERK inhibitor for 24 h and then stained with LIVE/DEAD, viability/cytotoxicity Kit from Thermo Fisher Scientific as described previously.^30^ Briefly, Live/dead stains were prepared, and cells were stained according to the kit protocol. Finally, cells were visualized, and images were captured using the EVOS FLc cell imaging system.

### 2.9 Cell lysis and immunoblotting

To investigate the underlying molecular mechanism of Glabridin‐mediated anti‐cancer actions in CTCL, cells were treated with Glabridin alone and or in combination with bortezomib/ERK inhibitor/ZVAD‐FMK, 3MA and so forth and immunoblotting was performed as described previously.^30^ Briefly, treated, and untreated cells were harvested and washed with PBS followed by cell lysis and determination of the protein concentration. Proteins were separated using SDS‐PAGE and transferred to polyvinylidene difluoride (PVDF) membrane followed by blocking and incubation with primary antibodies at 4°C overnight. Next morning, blots were washed and incubated with secondary antibodies and finally after washing blots were visualized under a Chemi‐Doc System (Bio‐Rad, Hercules, California, USA) using ECL.

### 2.10 Spheroid culture

Spheroids were generated in the lab in the form of spheres. Initially, cells were grown in normal 10% RPMI media. Then for the sphere generation, cells were cultured and treated in ultra‐low attachment plates/flasks (Corning, USA) using the complete cancer stem cell medium (3D Tumorsphere Medium XF, Promo Cell, Germany, C‐28070) at 37°C in a humidified chamber containing 5% CO_2_. Cells were monitored regularly and after 7 days, spheres were photographed using EVOS FL imaging system from Invitrogen (Thermo Fisher Scientific) 4× magnification.

### 2.11 Targeted metabolomics and data analysis

Metabolomic profiling of the CTCL cells treated with Glabridin was performed using the MxP® Quant 500 kit (Biocrates Life Sciences AG, Innsbruck, Austria) with an UHPLC‐MS/MS system, which consists of an ultra‐High‐Performance Liquid Chromatography (UHPLC) and a Triple Quad 5500+ MS/MS system (SCIEX, Framingham, MA, USA). Data were acquired from flow injection analysis (FIA), and liquid chromatography (LC) methods in both positive and negative ionization modes to accomplish the identification and quantification of metabolites according to the manufacturer's instructions as described previously.^31^ In brief, 10 μL samples (Cell Lysate),   calibration standards, quality control samples (provided with the kit) and zero samples were transferred to each predefined well  of the 96‐well filter plate that contains internal standards. Next, the kit plate was dried and incubated with 5% phenyl‐isothiocyanate to derivatize amino acids and biogenic amines in all the samples. Then, the plate with derivatized samples was dried using the Ultravap Mistral (Porvair Sciences) and analytes were eluted with 5 mmol/L ammonium acetate in methanol followed by dilution (two‐fold for LC–MS/MS and 50‐fold for FIA‐MS/MS) and finally the samples were analyzed by UHPLC‐MS/MS system.  We used MxP® Quant 500 kit LC column system (Biocrates) with the gradient mobile phases A: 0.2% formic acid in water and B: 0.2% formic acid in acetonitrile. All the parameters including the run time, ionization, voltages, temperatures, and the detection of m/z pair precursor and product ion at multiple reaction monitoring (MRM) mode were set as per the MxP® Quant 500 kit method. Data were recorded using the Analyst software (SCIEX) and transferred to the MetIDQ software (Biocrates) for further data processing. Further analysis, including data normalization, univariant and multivariant statistics, metabolite enrichment and pathway analysis was done using MetaboAnalyst 6.0 (https://www.metaboanalyst.ca/).^32^

### 2.12 Statistical analysis

Data from various experimental groups are presented as mean ± SD. Statistical calculations (ANOVA, post hoc test) were performed by using GraphPad Prism (GraphPad Software Inc., San Diego, CA, http://www.graphpad.com). Values of *p < 0.05 were considered statistically significant.

## 3 RESULTS

### 3.1 Glabridin inhibits CTCL cell viability

**FIGURE 1:**
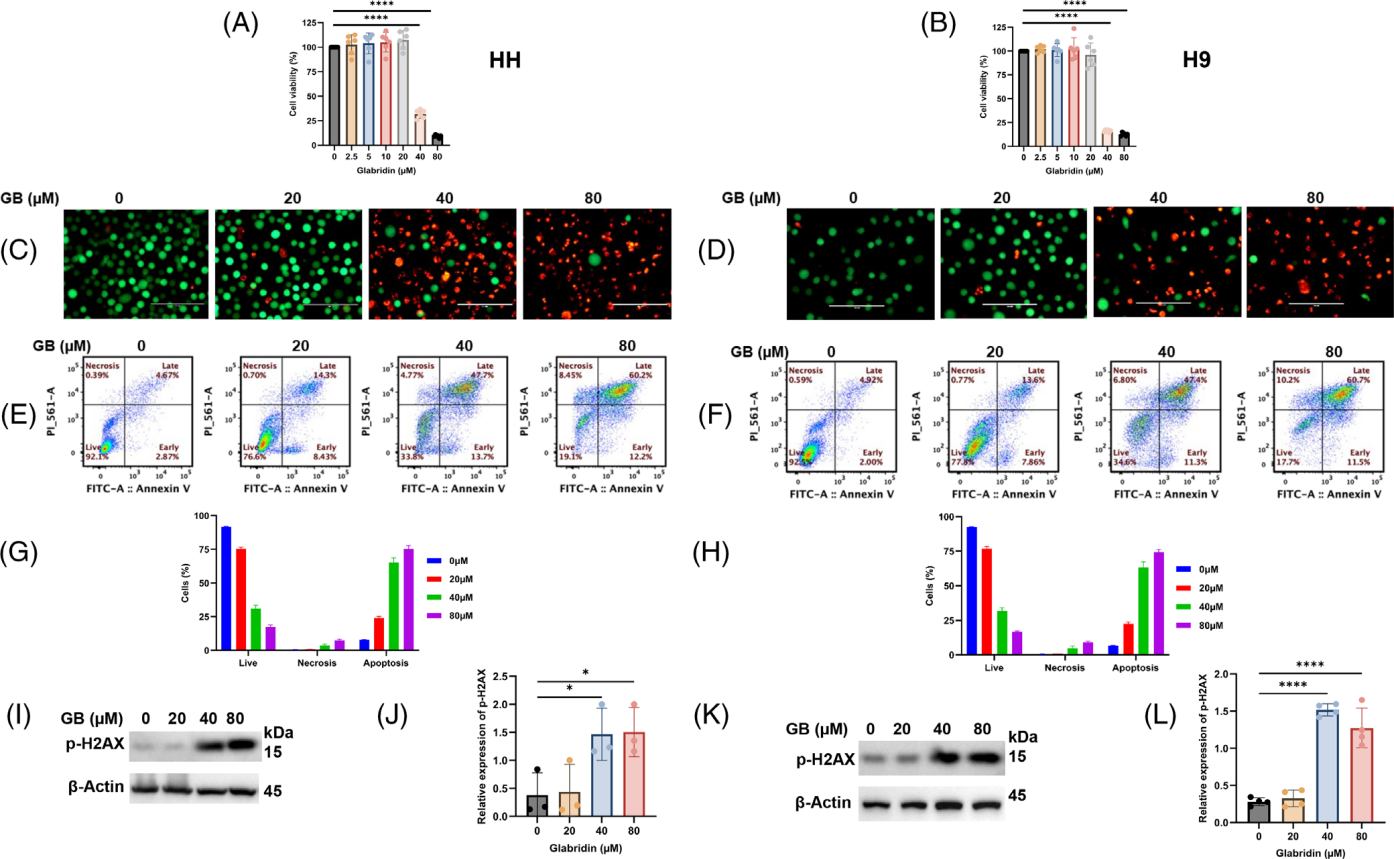
Glabridin suppressed the growth of CTCL cells. (A and B) The effect of the indicated concentration of Glabridin on the % cell viability of HH and H9 cells and data were presented as mean ± SD (*n* = 6). (C and D) The live and dead cell staining in Glabridin treated HH and H9 cells. For the identification of live cells, we have used green, fluorescent calcein‐AM dye for the intracellular esterase and red‐fluorescent ethidium homodimer‐1 which indicates a loss of membrane integrity (scale bar 100 μm; magnification 40×). (E and F) The representative results of HH and H9 cells treated with the indicated concentrations of Glabridin for 24 h followed by staining with fluorescein‐conjugated Annexin‐V/PI, and apoptotic as well as necrotic cells were determined by flow cytometry (*n* = 3). (G and H) The percentage of apoptosis and necrosis in cells of the treatment groups and the data is expressed as mean ± SD (*n* = 3). (I–L) Glabridin induced expression of DNA damage marker. HH and H9 cells were treated with Glabridin, and lysates were prepared. Detection of p‐H2AX by western blot analysis and its relative quantification was presented as mean ± SD (*n* = 3). The intensity of the bands was normalized with the respective loading control and quantified using image lab software. *p < 0.05 and ****p < 0.0001 represents the level of significance between treatment groups with respect to control.

**FIGURE 2:**
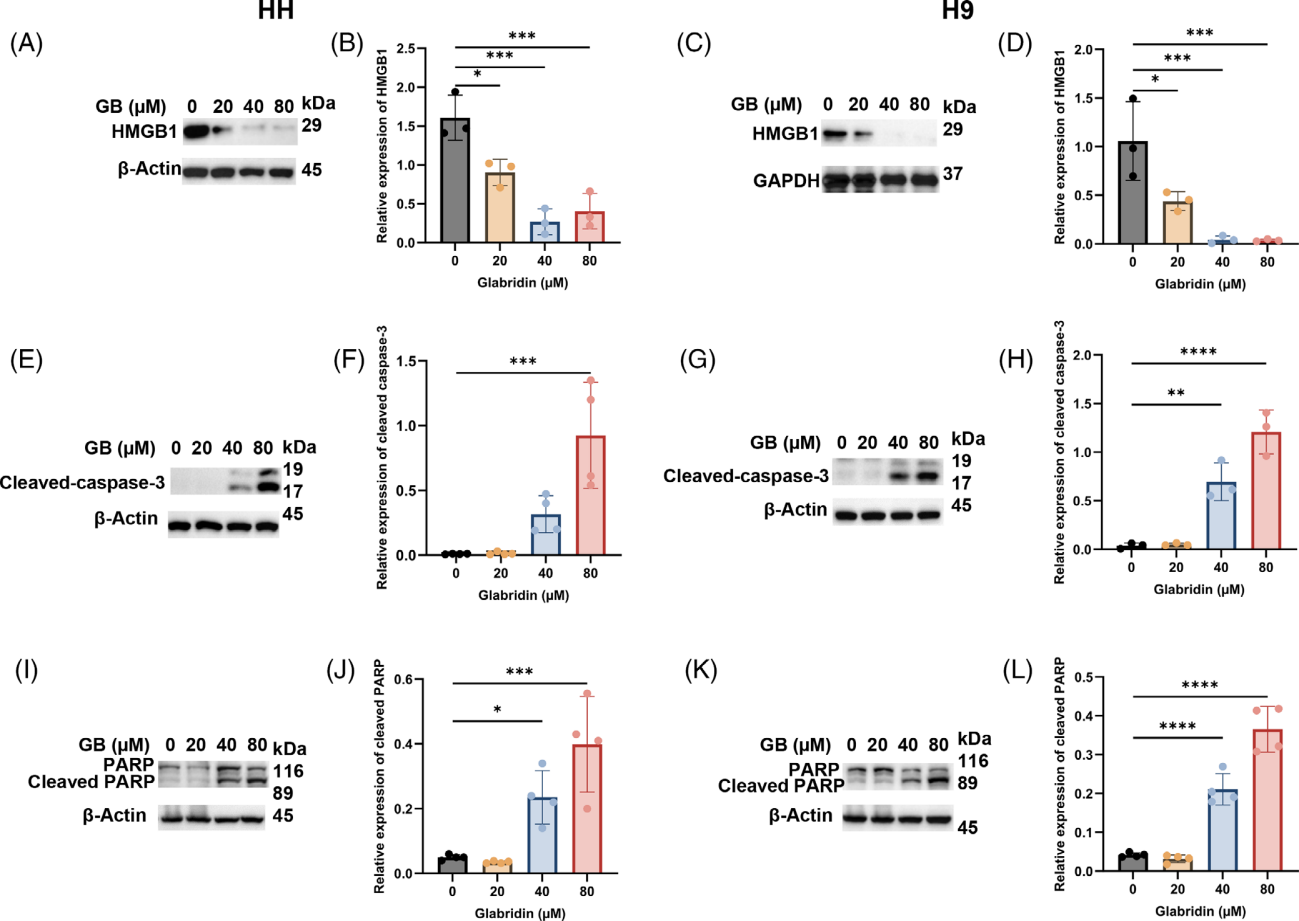
Glabridin induces cytotoxic effects through programmed cell death. CTCL cell lines, HH and H9 were treated with the indicated concentrations of Glabridin, and cell lysates were prepared and then immunoblotted. (A–L) Western blot analysis of three proteins (HMGB1, cleaved caspase‐3 and PARP) and their relative quantification result are presented as mean ± SD (*n* = 3). The intensity of the bands was normalized with the respective loading control and quantified using image lab software. *p < 0.05, **p < 0.01, ***p < 0.001 and ****p < 0.0001 represents the level of significance between treatment groups with respect to control.

Deregulated programmed cell death (PCD) is a key factor in orchestrating the acquisition of cancer hallmarks, and hence requires further investigation. In this line, we first treated the human CTCL cells with Glabridin at different concentrations (0, 2.5, 5, 10, 20, 40 and 80 μM) for 24 h, followed by CCK‐8 based cell viability test and interestingly our results showed a significant reduction in the percentage cell viability in CTCL cells (HH and H9) (*p* < 0.0001) (Figure 1A,B). Further, we also compared the cytotoxic effect of Glabridin with well‐known anti‐cancer drugs, doxorubicin and azacytidine (Supplementary Figure S1A,B). Next, we also observed a notable change in the intracellular (nucleus) as well as in the cell membrane as demonstrated by the live and dead cell staining in Glabridin treated HH (Figure 1C) and H9 (Figure 1D) cells. In line, Glabridin treatment significantly altered the cell cycle distribution pattern, which resulted in the accumulation of cells/cell arrest at the sub G0‐G1 phase in HH and H9 cells (Supplementary Figure S1C–F). Moreover, Glabridin also causes a significant decrease in mitochondrial membrane potential (MMP) in CTCL cells (Supplementary Figure S2A–D). Indeed, flow cytometry data of FITC Annexin V and PI staining, to identify cell death and determine apoptosis and necrosis, also showed that Glabridin treatment results in a significantly increased number of apoptotic and necrotic cells (Figure 1E–H) and hence supports its anti‐proliferative potential. Considering the growth inhibitory effects of Glabridin, we explored the associated underlying mechanism and our data showed activated p‐H2AX and HMGB1 (Figures 1I–L and 2A–D). HMGB1 (High Mobility Group Box 1 protein) is a ubiquitous non‐histone nuclear protein, passively released by the necrotic cells. Thus, these data indicate the potential of Glabridin as an inducer of PCD in CTCL cells.

### 3.2 Glabridin activates apoptosis in CTCL cells

Considering the strong anti‐proliferative potential of Glabridin, we explored the status of the regulatory proteins associated with apoptosis. Interestingly, Glabridin‐mediated apoptosis through upregulation/activation of cleaved caspase‐3 in CTCL cells as indicated in Figure 2E–H. Indeed, the expression of poly ADP ribose polymerase (PARP), a DNA damage repair enzyme, is also significantly altered by Glabridin in CTCL cells (Figure 2I–L).

Next, we used z‐VAD‐FMK, a pan‐caspase inhibitor to confirm the role of apoptosis in Glabridin‐induced CTCL cell death. As shown in Supplementary Figure S3A–H, z‐VAD‐FMK reversed the Glabridin‐induced apoptosis as evident from the expression of apoptotic markers and hence supports the crucial role of apoptosis in Glabridin treated CTCL cells.

### 3.3 Glabridin induces autophagy in CTCL cells

**FIGURE 3:**
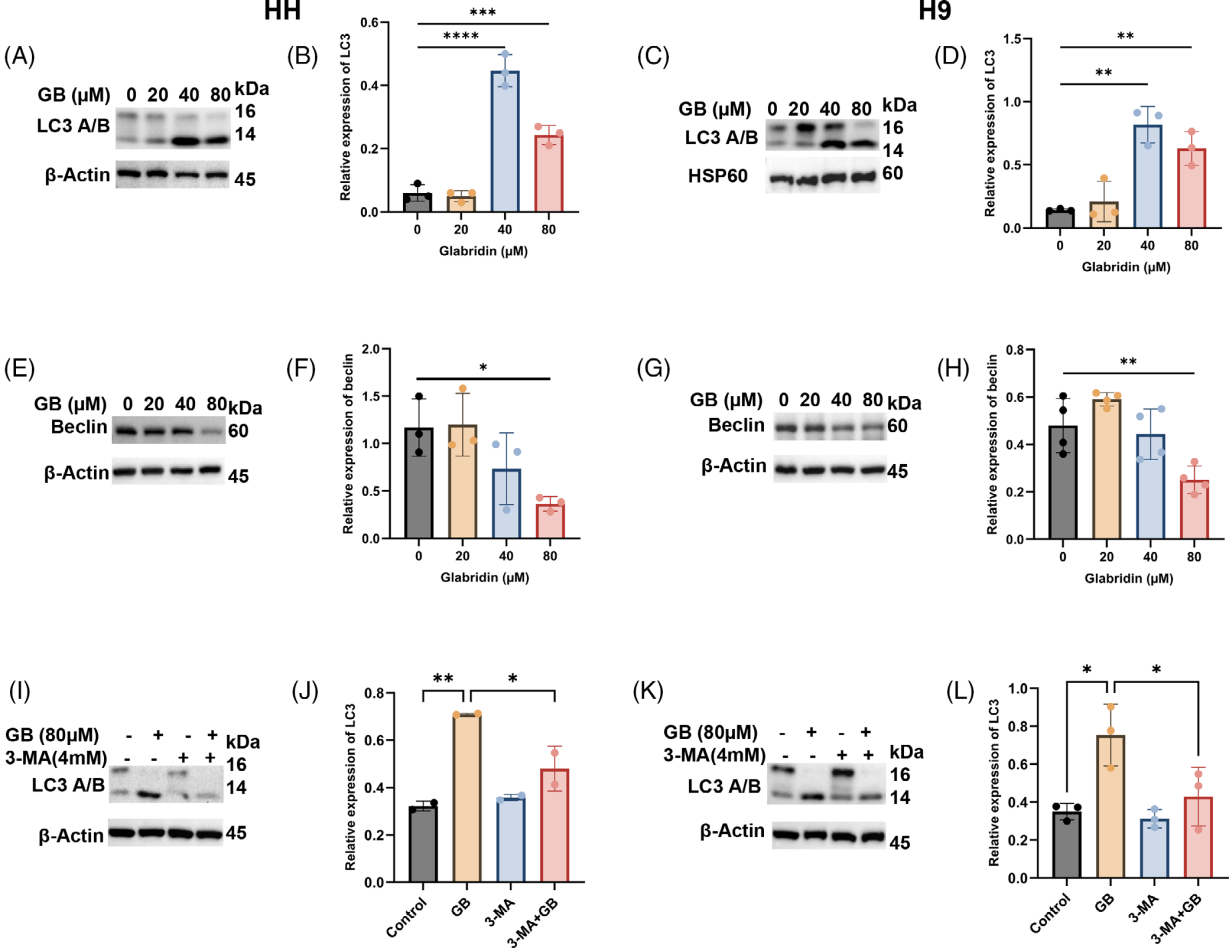
Glabridin treatment induces autophagy in CTCL cells. HH and H9 cells were treated with the indicated concentrations of Glabridin, and cell lysates were prepared and immunoblotted against autophagy markers. (A–H) Western blot analysis of LC3 and beclin and their relative quantification result are presented as mean ± SD (*n* = 3). The intensity of the bands was normalized with the respective loading control and quantified using the image lab software. (I–L) HH and H9 cells were treated with the indicated concentration of Glabridin in the presence and absence of 3‐methyladenine (3‐MA) and cell lysates were prepared followed by expression analysis of LC3. Western blot analysis of LC3 and its relative quantification results are presented as mean ± SD (*n* = 3). The intensity of the bands was normalized with the respective loading control and quantified using image lab software. *p < 0.05, **p < 0.01, ***p < 0.001 and ****p < 0.0001 represent the level of significance between treatment groups relative to control groups.

Autophagy, another type of PCD, plays a crucial role in cellular and biological homeostasis, often supporting cancer cell survival and growth. Keeping this in mind, we explored the role of autophagy in Glabridin‐treated CTCL cells. Indeed, our data show that Glabridin induces upregulation of autophagy, as evident from LC3 expression (Figure 3A–D). In line, our result also revealed that Glabridin causes modulation of autophagy regulatory proteins in CTCL cells (Figure 3E–H). Importantly, 3‐Methyladenine (3‐MA), an autophagy inhibitor, treatment reversed Glabridin mediated autophagy in CTCL cells and thus indicated the role of autophagy (Figure 3I–L). Further, we also checked the status of the apoptotic markers, caspase‐3 and cleaved caspase‐3 (Supplementary Figure S3I–N) and observed an increase in capase‐3 activation in CTCL cells treated with 3‐MA and Glabridin. Thus, these results suggest that Glabridin induced autophagy could repress apoptosis in Glabridin treated CTCL cells, or in other words Glabridin treatment causes protective/antiapoptotic autophagy in CTCL cells and hence autophagy inhibition may enhace the apoptotic effects of Glabridin in CTCL cells.

### 3.4 Glabridin modulates MAPK signalling in CTCL cells

**FIGURE 4:**
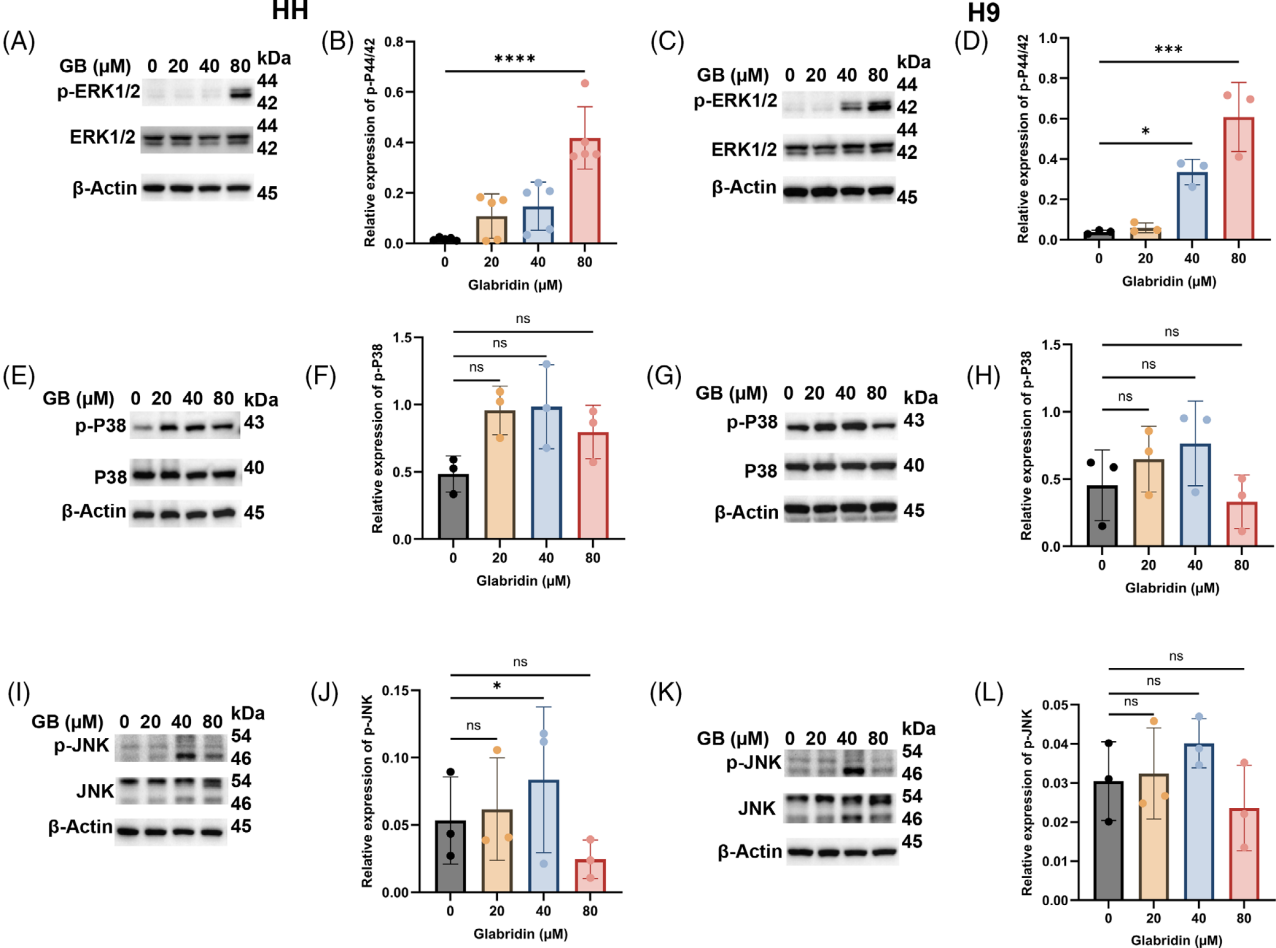
Glabridin modulates MPAK signalling pathways in CTCL. HH and H9 cells were treated with indicated concentrations of Glabridin followed by lysate preparation and immunoblotting. (A–D) Western blot analysis of p‐P44/42, P44/42 and their relative quantification results are presented as mean ± SD (*n* = 3). The intensity of the bands was normalized with the β‐actin and quantified using image lab software. (E–H) Western blot analysis of p‐P38, P38 and their relative quantification results are presented as mean ± SD (*n* = 3). The intensity of the bands was normalized with the respective loading control and quantified using image lab software. (I–L) Western blot analysis of p‐PJNK, JNK and their relative quantification results are presented as mean ± SD (*n* = 3). The intensity of the bands was normalized with the respective loading control and quantified using image lab software. *p < 0.05, ***p < 0.01 and ****p < 0.0001 represent the level of significance between treatment groups relative to control. ns, non‐significant.

MAPK signalling is imperative in a myriad of biological functions from the developmental stage to the growing phase and till death. Interestingly, these pathways are very much involved in the repair and prolong survival of cancer cells which often translate into cancer hallmarks such as drug resistance and limitless proliferation. Importantly, cancer therapeutic measures including phototherapy and chemotherapy also act through these pathways. Hence, we checked the role of MAPK in Glabridin‐induced anti‐cancer actions in CTCL cells. In this line, our results clearly show that Glabridin modulated the expression and the function of MAPK signalling. As shown in Figure 4, Glabridin markedly modulated the expression of MAPK signalling pathways (ERK, p38 and JNK). However, our results showed marked ERK activation at 40 and 80 μM concentrations of Glabridin treatments in CTCL cells (Figure 4A–D). Moreover, we did not detect a significant change in the expression of total ERK proteins. Interestingly, Glabridin induced activation of p‐P38 and p‐JNK was not consistent with its increasing concentrations (Figure 4E–L). Thus, our results suggest that Glabridin‐induced modulation of MAPK pathways particularly ERK is crucial and potentially the important underlying mechanism of Glabridin.

### 3.5 Glabridin induces programmed cell death in CTCL cells through ERK activation

**FIGURE 5:**
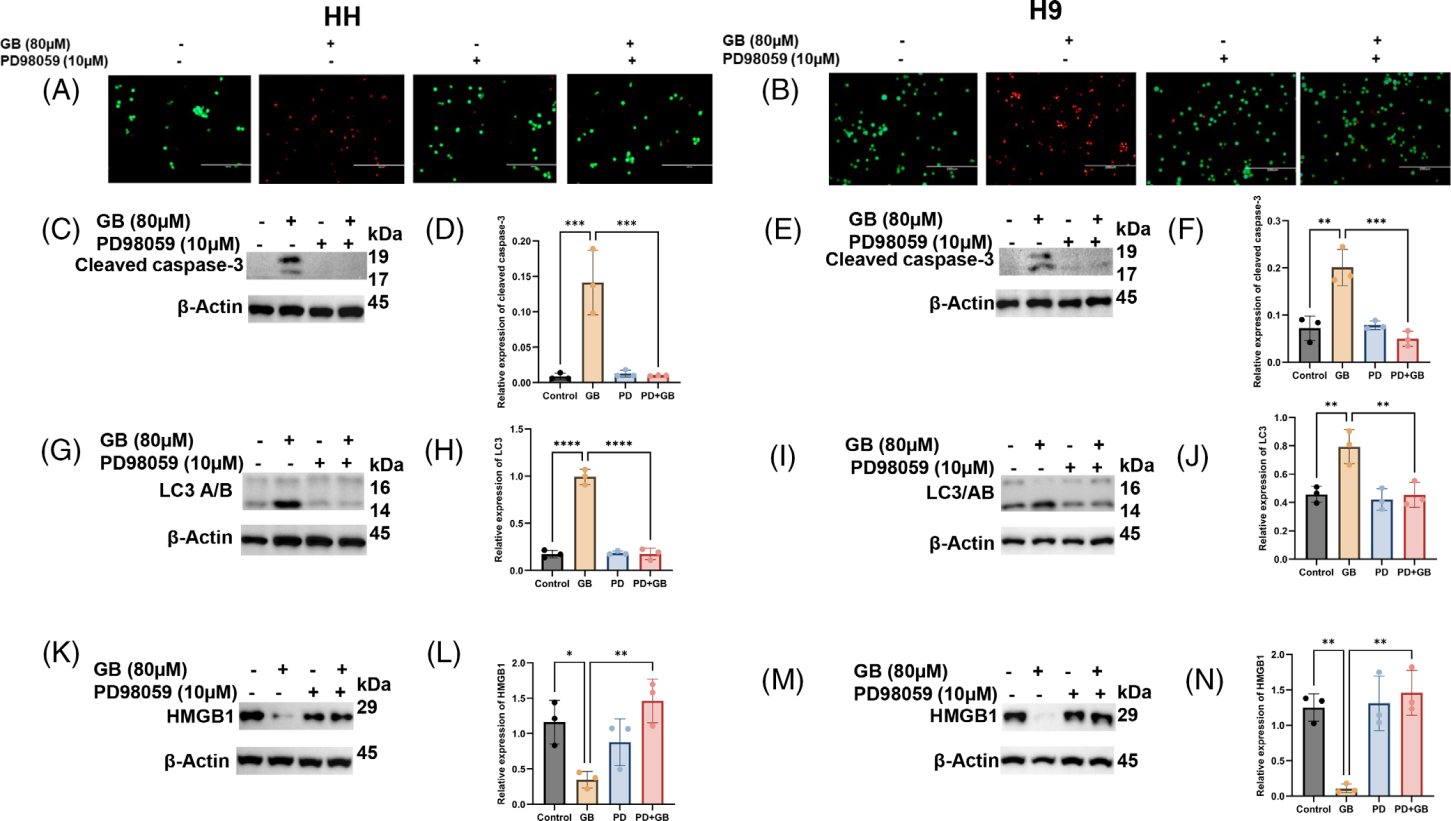
ERK inhibition prevents Glabridin induced PCD in CTCL cells. HH and H9 cells were treated with the indicated concentration of Glabridin and or PD98059 alone and in combination. (A and B) The live and dead cell staining HH and H9 cells. For the identification of live cells, we have used green fluorescent calcein‐AM dye for the intracellular esterase and red‐fluorescent ethidium homodimer‐1 which indicates a loss of membrane integrity (scale bar 200 μm; magnification 20×). (C–N) HH and H9 cells were treated with the indicated concentration of Glabridin and or PD98059 alone and in combination and cell lysates were prepared followed by immunoblotting. Western blot analysis of cleaved caspase 3, LC3 and HMGB1 and their relative quantification results are presented as mean ± SD (*n* = 3). The intensity of the bands was normalized with the respective loading control and quantified using image lab software. *p < 0.05, **p < 0.01, ***p < 0.001 and ****p < 0.0001 represent the level of significance between treatment groups relative to control (positive and negative) groups.

ERK signalling is vital for the maintenance of cell growth and proliferation, and protection from endogenous as well as exogenous insults. Additionally, ERK activation is very much involved in cancer therapeutic measures, including phototherapy and chemotherapy. Hence, we wanted to explore whether Glabridin induced ERK activation is crucial in orchestrating the PCD in CTCL cells or not. To do so, we treated CTCL cells with PD98059, a selective inhibitor of ERK1/2 signalling, alone and in combination with Glabridin and studied various parameters. In this line, our data from live and dead cell staining showed that PD98059 markedly reversed Glabridin‐induced cell death, as shown in Figure 5A,B. Next, PD98059 mediated ERK inhibition markedly reversed the number of cells in the apoptotic and necrosis stages due to Glabridin treatment in CTCL cells (Supplementary Figure S4A). This further supports the crucial role of ERK in Glabridin‐mediated anti‐cancer actions in CTCL cells. Moreover, we also checked the underlying mechanisms associated with PCD in CTCL cells treated with PD98059 alone and in combination with Glabridin. Intriguingly, the results showed a strong association of apoptosis, autophagy and necrosis with Glabridin‐induced ERK activation in CTCL cells. As shown in Figure 5C–N, ERK inhibition markedly reversed the expression of proteins associated with PCD due to Glabridin treatment in CTCL cells. Thus, these results demonstrated that Glabridin induced PCD in CTCL cells is ERK‐dependent.

### 3.6 Metabolomics profiling of CTCL cells treated with Glabridin and ERK inhibitor PD98059

**FIGURE 6:**
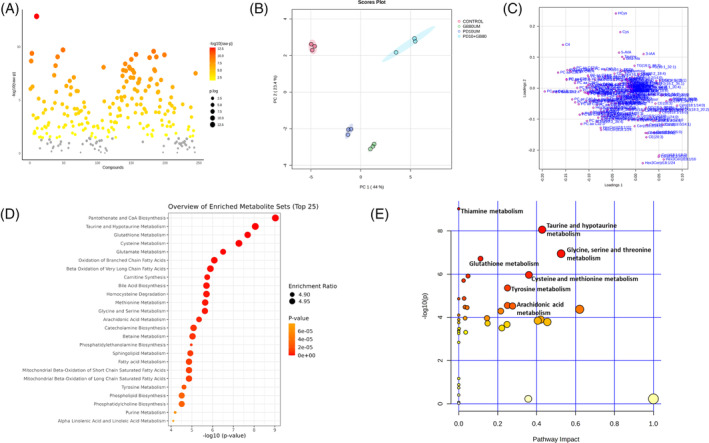
Metabolomic profiling of CTCL cells (H9) treated with Glabridin and ERK inhibitor PD98059. (A) Scatter plot showing the important features identified by ANOVA plot with −log(10)*p* values on the y‐axis and metabolites (compounds) on the x‐axis. Coloured dots show the significant metabolites with a *p*‐value <0.05. (B) Principal component analysis (PCA) of targeted metabolomics data from the samples of different experimental groups (control, Glabridin 80 μM, PD10μM and PD10μM+ Glabridin 80 μM) of CTCL cells. (C) Loading plot of the first two principal components. Functional analysis of the significant features in Glabridin treated CTCL cells using MetaboAnalyst 6.0 (https://www.metaboanalyst.ca/). (D) Quantitative enrichment analysis (QEA) overview presenting the top 25 metabolic pathways. Within a particular metabolic pathway, enrichment ratio is calculated as the number of observed hits/expected hits. (E) Metabolome view of the important metabolic pathways. The pathway impact values (x‐axis) represent the influencing factor of a topological analysis, and the –log(*p*) (y‐axis) represents the *p*‐value of the pathway enrichment analysis. Each circle represents a pathway, and the colour and size of each circle are based on the *p*‐value of the pathway enrichment analysis and pathways impact values from the pathway topology analysis, respectively.

LCMS‐based targeted metabolic profiling was done using the MxP Quant 500 kit (biocrates) to understand the metabolic alterations in CTCL cells treated with Glabridin and ERK inhibitor PD98059. Data normalization and statistical analysis (univariant and multivariant) were done using Metaboanalyst 6.0.^32^ One‐way ANOVA with post hoc analysis identified 186 significantly altered features in CTCL cells treated with Glabridin and PD98059 alone and in combination (Figure 6A; Supplementary Table S1). Statistical results including *f*‐values, *p*‐values and FDR values of the significant features are mentioned in Supplementary Table S1. A heatmap analysis of the significant features (*p* < 0.05) also showed the differential level in various treatment groups as shown in Supplementary Figure S5A. Red represents a high concentration level of metabolite and green represents a low metabolite concentration level (Supplementary Figure S5A). We further performed principal component analysis (PCA) which showed a clear separation of each group with a variance of the first principal component (PC1) (44%), and second principal component (PC2) (23.4%) (Figure 6B) thus demonstrating a distinct metabolic signature or a differential metabolism of cells in each treatment group. The corresponding 2D loading plot is also shown in Figure 6C. ANOVA with post hoc analysis shows the significant modulation in different categories of metabolites, including amino acid, amino acid related, biogenic amines, carboxylic acids, fatty acid, ceramides, hexosylceramides, acylcarnitines, nucleobases related and vitamins and cofactors, in Glabridin treated CTCL cells in an ERK‐dependent fashion (Supplementary Figure S6A–F, Supplementary Table S1). Level of six amino acids (aspartic acid [Asp], cysteine [Cys], glycine [Gly], glutamic acid [Glu], alanine [Ala] and threonine [Thr]) and nine amino acid related metabolites (asymmetric dimethylarginine [ADMA], 5‐aminovaleric acid [5‐AVA], citruline, cystine, levodopa or dihydroxyphenylalanine [DOPA], homocysteine [Hcys], 3‐methylhistidine [3‐Met‐His], trans‐4‐hydroxyproline [t4‐OH‐Pro] and Taurine) was significantly modulated due to Glabridin treatment and interestingly, level of all these metabolites was reversed by ERK inhibition (Supplementary Figure S6A,B and Supplementary Table S1).

Fatty acids are another important category of metabolites strongly associated with cancer pathogenesis. Metabolic profiling data revealed that arachidonic acid (AA), docosahexaenoic acid (DHA), FA(20:3) (eicosatrienoic acid) and FA(18:2) (octadecadienoic acid) level was significantly altered by Glabridin which was reversed by ERK inhibition (Supplementary Figure S6C, Supplementary Table S1). In addition, four carboxylic acids namely, aconacid (aconitic acid), Suc (succinic acid), Lac (lactic acid) and OH‐Glut acid (3‐hydroxyglutaric acid) level were significantly reduced by Glabridin and was reversed due to ERK inhibition (Supplementary Figure S6D, Supplementary Table S1). Moreover, metabolites of different categories such as biogenic amines (spermine, gamma‐aminobutyric acid [GABA] and β‐alanine) (Supplementary Figure S6D, Supplementary Table S1), ceramides and hexocylceramides (Supplementary Figure S6E, Supplementary Table S1), acylcarnitines, choline and hypoxanthine (Supplementary Figure S6F, Supplementary Table S1), was significantly modulated by Glabridin in an ERK dependent manner. Hypoxanthine, a nucleobase purine derived metabolite, was also significantly reduced by Glabridin in CTCL cells (Supplementary Figure S6F, Supplementary Table S1).

Moreover, we also performed metabolite enrichment and pathway analysis of the significant features (*p* < 0.05), based on the Small Molecule Pathway Database (SMPDB) database and Kyoto Encyclopedia of Genes and Genomes (KEGG) database using Metaboanalyst 6.0. respectively. Interestingly, quantitative enrichment analysis of the significant metabolites in Glabridin treated CTCL cells shows the enrichment of the significant features in several important metabolic pathways, as shown in Figure 6D. Importantly, pathway analysis reveals a total of 41 metabolic pathways modulated in Glabridin treated CTCL cells (Figure 6E; Supplementary Table S2). Importantly, thiamine metabolism, taurine and hypotaurine metabolism, glycine, serine and threonine metabolism, glutathione metabolism and cysteine and methionine metabolism are the major five metabolic pathways affected in Glabridin treated CTCL cells (Figure 6E). Inline, various statistical nomenclatures, including *p*‐value, −log(*p*) value, Holm‐*p* value, false discovery rate (FDR) value and pathway impact values are mentioned in the Supplementary Table S2. Similarly, an overview of metabolite set enrichment analysis of the significant features in Glabridin and PD98089 + Glabridin is shown in Supplementary Figure S5B. Further, pathway analysis of Glabridin and PD98089 + GB treated cells show that thiamine metabolism, glycine, serine and threonine metabolism, glutathione metabolism, taurine and hypotaurine metabolism, and cysteine and methionine metabolism, are the five most significantly affected metabolic pathways as shown in Supplementary Figure S5C and Supplementary Table S3.

### 3.7 Glabridin targets signalling pathways associated with metabolic reprogramming through ERK activation

**FIGURE 7:**
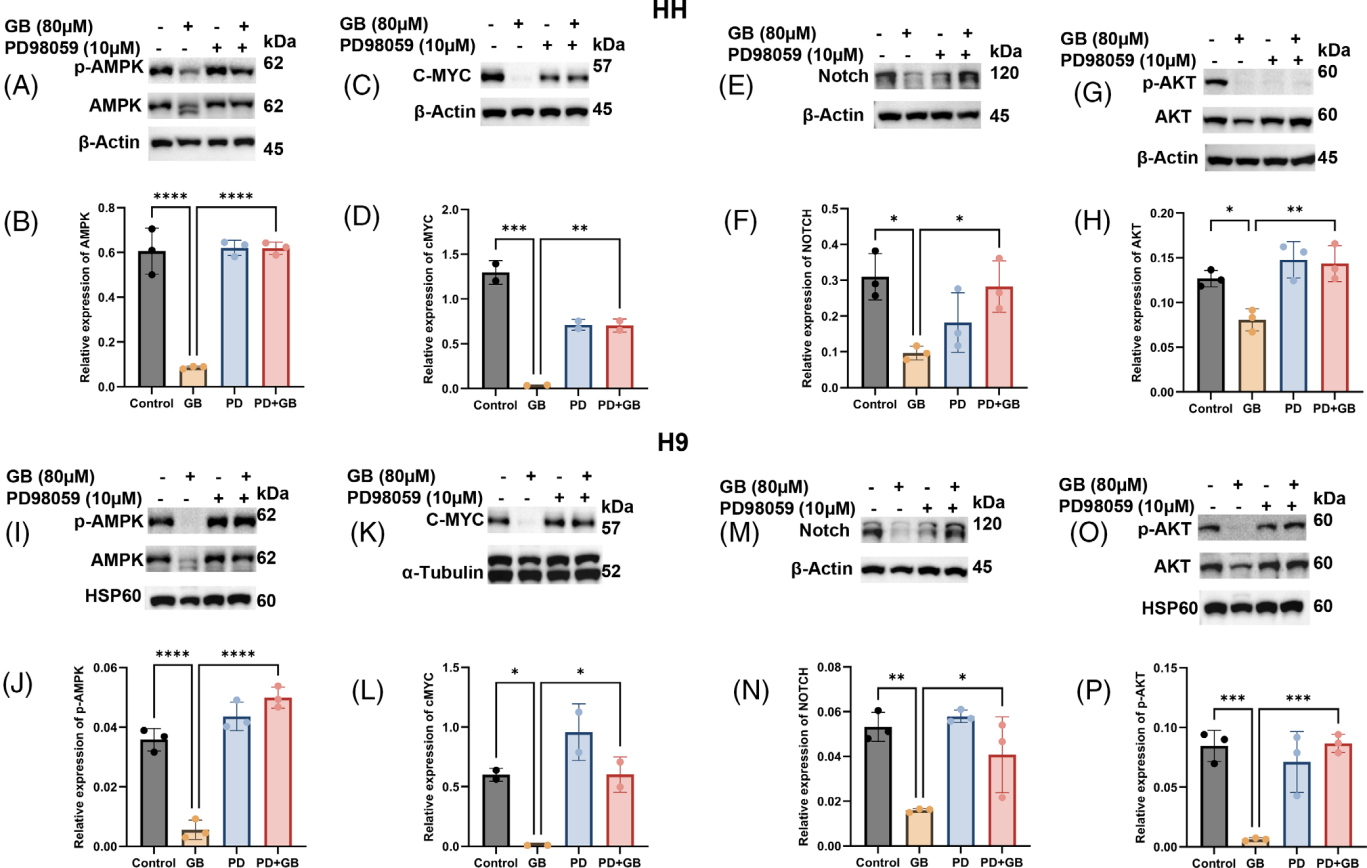
Glabridin targets signalling pathways associated with metabolic reprogramming through ERK. HH and H9 cells were treated with the indicated concentrations of Glabridin and PD98059 alone and in combination followed by cell lysates preparation and immunoblotting. (A–P) The western blot analysis of p‐AMPK, AMPK, c‐MYC, Notch, p‐AKT and AKT and their relative quantification results are presented as mean ± SD (*n* = 2, *n* = 3). The intensity of the bands was normalized with the respective loading control and quantified using image lab software. *p < 0.05, **p < 0.01, ***p < 0.001 and ****p < 0.0001 represent the level of significance between treatment groups relative to control (positive and negative) groups.

Deregulated metabolism is a complex and heterogenic phenomenon regulated by a range of signalling mechanisms and pathways. Increasing evidence indicates that cancer cells accomplish metabolic reprogramming through various deregulated signalling mechanisms and thus ultimately acquire different hallmarks including drug resistance and poor clinical outcomes. Therefore, targeting multiple pathways and regulatory molecules associated with metabolic reprogramming is imperative. In this line, we found that Glabridin markedly inhibited the expression of major signalling pathways such as AMPK, C‐MYC, AKT and NOTCH (Supplementary Figure S7A–P) associated with metabolic reprogramming in cancer cells. Next, we further explored the role of ERK in the Glabridin‐induced inhibition of these signalling pathways. Interestingly, as shown in Figure 7A–P, inhibition of ERK with PD98059 markedly reversed the expression of the above‐mentioned signalling proteins in Glabridin treated CTCL cells. Hence, these findings indicate the ERK dependent multi‐targeting potential of Glabridin in CTCL cells. Interestingly, we also found that Glabridin inhibits spheroid formation in CTCL cells in an ERK dependent fashion (Supplementary Figure S4B,C).

### 3.8 Glabridin sensitized CTCL cells to bortezomib

**FIGURE 8:**
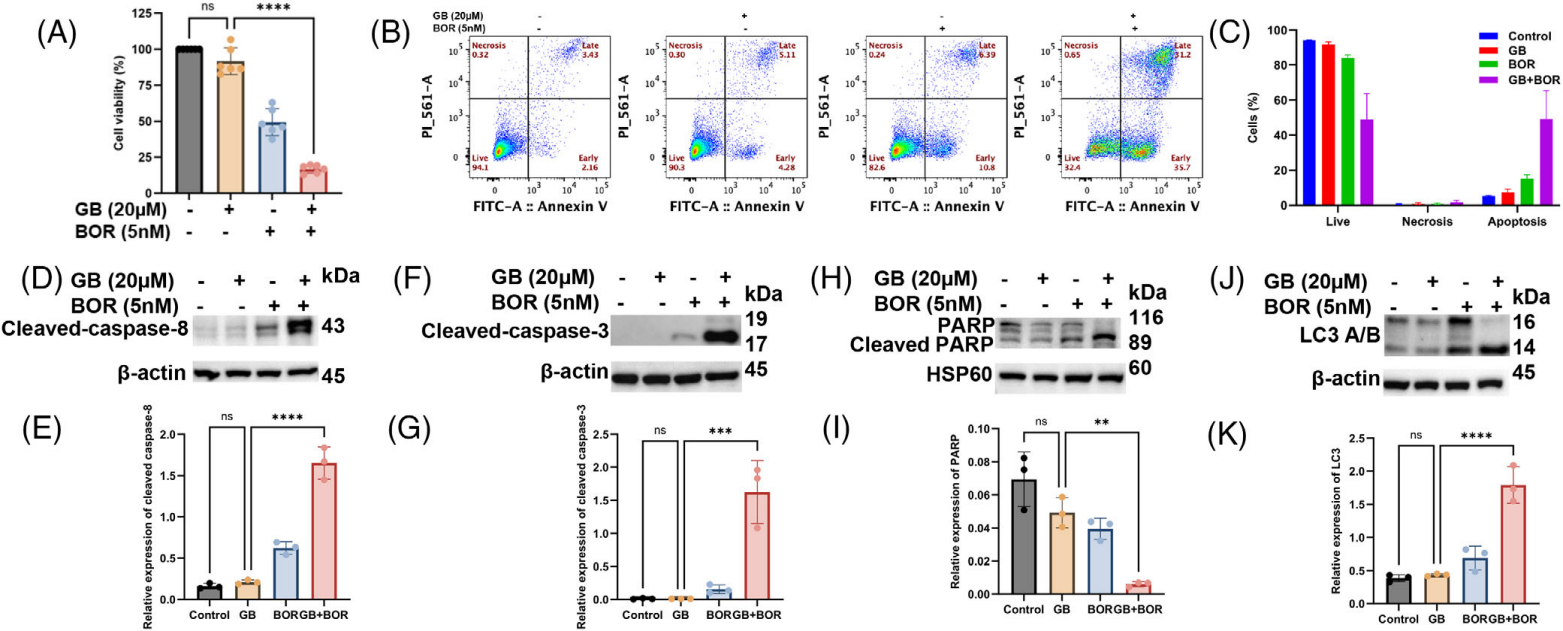
Glabridin sensitizes CTCL cells to bortezomib. (A) The effect of the indicated concentration of Glabridin and bortezomib alone and in combination on the % cell viability of H9 cells. Results are expressed as mean ± SD (*n* = 6). (B) The representative data of H9 cells treated with the indicated concentration of Glabridin and bortezomib alone and in combination for 24 h followed by staining with fluorescein‐conjugated Annexin‐V/PI, and apoptotic and necrotic cells were determined by flow cytometry. (C) The percent of apoptosis and necrosis in cells of different treatment groups and the data is expressed as mean ± SD (*n* = 3). (D–K) The western blot analysis of cleaved caspase‐8, cleaved caspase‐3, PARP, and LC3, and their relative quantification results are presented as mean ± SD (*n* = 3). The intensity of the bands was normalized with the respective loading control and quantified using image lab software. **p < 0.01, ***p < 0.001 and ****p < 0.0001 represent the level of significance between treatment groups relative to control (positive and negative) groups.

Chemoresistance or resistance to anti‐cancer drugs is a major challenge of cancer therapy and hence requires the exploration of novel and effective measures. Intriguingly, we investigated the sensitizing potential of Glabridin towards bortezomib in CTCL cells. As shown in Figure 8A, the CCK‐8 assay reveals a significant decrease in cell viability in Glabridin + bortezomib treated cells as compared to only Glabridin treatment. Further, flow cytometry data depicted that Glabridin treatment sensitizes CTCL cells to bortezomib as there is a significant increase in the percentage of apoptotic cells in Glabridin + bortezomib treated cells as compared to their alone counterpart (Figure 8B,C). Furthermore, our results also showed increased expression of apoptotic and autophagy markers in CTCL cells treated with Glabridin in combination with bortezomib as compared to alone treatments (Figure 8D–K). Hence, based on these results, it can be postulated that Glabridin has strong sensitizing potential to anti‐cancer drugs and thus has the potential of a suitable sensitizing agent.

## 4 DISCUSSION

CTCL is an increasing health‐related concern and hence, understanding the underlying mechanism associated with CTCL pathogenesis is imperative to improve the patient's health. There is growing concern about re‐investigating the role of MAPK signalling in regulating cell survival and the stemness of cancer cells. Hence in the current study for the first time, we have explored the crucial roles of MAPK pathways in regulation of the underlying mechanisms and metabolic pathways related to the orchestration of cancer hallmarks in CTCL cells treated with Glabridin.

Furthermore, there is growing concern about developing novel and effective treatment regimens with no or very minimal adverse effects. In this line, much attention has been given to natural products due to their multi‐targeting nature and thus can attenuate the expression of major signalling pathways associated with cancer cell growth and stemness features. Glabridin, an active constituent of licorice (*G. glabra*) possesses a range of pharmacological actions, act through modulating various signalling pathways associated with cell proliferation, metabolism and programmed cell death.^24^, ^29^ Indeed, the data of the current investigation demonstrated that Glabridin inhibits the growth and proliferation of human CTCL cells through PCD, which includes apoptosis, autophagy and necrosis via targeting the deregulated oncogenic signalling pathways. Interestingly, Glabridin at 40, 80 and 100 μM have already shown similar pharmacological actions (anti‐tumour effect) and thus supports the findings of this study.^33^, ^34^, ^35^, ^36^

MAPK signalling is one of the most important mechanisms that regulates the developmental and physiological pathways and thus maintains cellular biological homeostasis. Interestingly, the results of this study show that ERK/MAPK is the major signalling target of Glabridin which agrees with the previous reports.^33^, ^36^ Indeed, inhibition of ERK with PD98059 supports its role in Glabridin‐induced programmed cell death in CTCL cells.

Targeting the PCD, which is the most deregulated mechanism in cancer cells, is an important strategy for the selective elimination of neoplastic cells. However, deregulation of apoptosis, autophagy and necrosis also play a key role in maintaining the cancer hallmarks, including cancer stemness and disease recurrence.^37^ Importantly, Glabridin mediated anti‐cancer actions through apoptosis, autophagy and necrosis as supported by the outcomes of this study. Further, we also highlight the crucial role of ERK in Glabridin induced PCD of human CTCL cell lines. Importantly, Glabridin treatment also attenuates the metabolic reprogramming and stemness properties of CTCL cells through MAPK signalling pathways which agrees with earlier reports.^38^, ^39^

Metabolic/energy reprogramming in cancer cells is the major driving force for the uncontrolled growth, proliferation and progression of cancer cells through escaping/defeating various regulatory mechanisms including immune escape from the neighbouring non‐cancerous cells like stromal immune cells.^40^ Hence targeting metabolic pathways linked with cancer cells as well as normal neighbouring cells would be an important strategy for the effective elimination of cancer cells and the associated tumour microenvironment.^40^ Interestingly, our metabolic profiling data indicates that Glabridin treatment markedly affected crucial metabolic pathways integral in CTCL pathogenesis and metabolic reprogramming. Moreover, metabolites, including amino acid, biogenic amines, amino acid related metabolites, fatty acids, carboxylic acids, ceramide, acylcarnitines and nucleobases and cofactors modulated by Glabridin in an ERK dependent manner suggesting an important association between cancer cell signalling and metabolic pathways. Similarly earlier report on metabolomics analysis shows that Glabridin treatment reversed the metabolic alterations induced by lipopolysaccharide (LPS) in macrophage cells.^41^

Amino acids (AA) and their related metabolites are the essential metabolites for the synthesis of various macromolecules including protein, lipids and nucleic acids and their upregulated level has a strong association with cancer pathobiology.^42^ Interestingly, Glabridin treatment causes increased level of fatty acids in CTCL cells which could be a potential trigger for the induction of PCD and is also supported by studies on fatty acid mediated cell death.^43^, ^44^, ^45^, ^46^ Ceramides are lipids (sphingolipids) play crucial role in cell proliferation programmed cell death and metabolic reprogramming.^47^, ^48^ Interestingly, anti‐cancer agents induce PCD in cancer cells through ceramide induction which corroborates with our findings.^49^ Dysregulated choline play a major role in cancer pathobiology, progression and stemness features including resistance.^50^, ^51^ Metabolic profiling of Glabridin treated CTCL cells showed depletion in choline level in an ERK dependent manner which is supported by others.^4^, ^52^

In this line, adenosine 5′‐monophosphate (AMP)‐activated protein kinase (AMPK) is the major protein that senses and regulates cellular energy balance through monitoring energy metabolic pathways and biological functioning of mitochondria and lysosomes and thus maintains cell growth and homeostasis.^53^, ^54^ Its role in cancer pathogenesis is not clear, as studies have shown its protumorigenic as well as anti‐tumorigenic potential.^55^, ^56^, ^57^ Moreover, AMPK activation is one of the main culprits in the growth and stemness of cancer cells via metabolic reprogramming.^58^, ^59^, ^60^ Hence, targeting AMPK could be an important measure for cancer management. Glabridin inhibits AMPK in human CTCL cells and thus supports its importance in modulating energy sensing metabolic pathways. Similarly earlier reports also revealed that AMPK inhibition suppresses cancer pathogenesis and sensitizes cancer cells to apoptosis.^60^, ^61^, ^62^ Furthermore, it has been also reported that AMPK inhibition promotes apoptosis in acute leukaemia to multiple BH3 mimetics.^63^ Beside AMPK, recent updates also highlight the role of c‐MYC, NOTCH and AKT signalling in the metabolic reprogramming of neoplastic cells.^20^, ^64^, ^65^, ^66^, ^67^ In line, Glabridin regulates these signalling pathways associated with metabolic rewiring in cancer cells through ERK activation thus supporting additional mechanisms contributing to metabolic reprogramming in cancer cells which corroborate with earlier findings.^26^

There has been growing concern about the limitations of the current treatment options for CTCL. Adverse side effects, poor prognosis and disease recurrence are the major challenges of the clinics. In this line, a combinational therapeutic approach has been given importance for better disease management. Indeed, our results further strengthened that Glabridin also sensitized the CTCL cells to bortezomib, a drug used for multiple myeloma. Importantly, Glabridin has shown no significant cytotoxic effects on normal human cells and thus indicates its potential anti‐cancer importance.^33^

In conclusion, mechanistic investigation as well as metabolomics profiling of Glabridin treated human CTCL cells showed a crucial role of ERK dependent pathways in regulating growth and proliferation. Thus, Glabridin through its multi‐targeting nature has shown promising outcomes that can be translated for the management of CTCL patients.

## AUTHOR CONTRIBUTIONS

**Khan AQ**: Study design, supervision, conceptualization, methodology, data collection and analysis, writing original draft, review and editing. **Agha MV**, **Anvar R**, **Khalid SA**: Data collection. **Fareed A**, **Mateo J**: Flow cytometry experiments and analysis. **Alam M**, **Buddenkotte J**, **Uddin S**: Visualization, writing—review and editing. **Steinhoff M**: Supervision, conceptualization, study design, writing—review and editing and resources. All authors gave their final approval and agree to be accountable for all aspects of the work.

## CONFLICT OF INTEREST STATEMENT

The authors declare no conflicts of interest.

## Supporting information


**Supplementary Figure S1.** Effect of Glabridin, doxorubicin (DOX) and azacytidine (AZA) on cell viability. (A) HH and (B) H9 cells were treated with the indicated concentration of GB, DOX and AZA for 24 h. CCK‐8 was used to determine the cell viability and data were presented as mean ± SD (*n* = 6). Effect of Glabridin on cell cycle distribution. (C and D) HH and (E and F) H9 cells were treated with different concentrations (0 μM, 20 μM, 40 μM, 80 μM), and the cell cycle distribution percentage was analysed by flow cytometry. Glabridin treatments induce a mark increase in the percentage of cells at the subG0‐G1 and G0‐G1 phase as compared to the control group in both HH (A and B) and H9 (C and D) cells. Results are presented as mean ± SD (*n* = 3).

**Supplementary Figure S2.** Effect of Glabridin on the mitochondrial membrane potential. HH and H9 cells were treated with different concentrations (0 μM, 20 μM, 40 μM, 80 μM), and mitochondrial membrane potential was analysed by flow cytometry. Glabridin treatment induces a marked increase in the loss of mitochondrial membrane potential as compared to the control group in both HH (A and B) and H9 (C and D) cells. Results are presented as mean ± SD (*n* = 3).

**Supplementary Figure S3.** Effect of Z‐Vad‐FMK on Glabridin induced apoptosis. HH and H9 cells were treated with the indicated concentration of Z‐VAD‐FMK and Glabridin (GB) alone in combination and then lysates were prepared, and immunoblotting was performed. (A–H) Western blot analysis of PARP, cleaved PARP and p‐H2AX and their relative quantification results are presented as mean ± SD (*n* = 3). (I–N) HH and H9 cells were treated with the indicated concentration of Glabridin in the presence and absence of 3‐MA and cell lysates were prepared followed by expression analysis and quantification of caspase‐3 and cleaved caspase‐3. The intensity of the bands was normalized with the respective loading control and quantified using image lab software. *p < 0.05 and **p < 0.01 represent the level of significance between treatment groups relative to control (positive and negative) groups.

**Supplementary Figure S4.** (A) The data of H9 cells treated with the indicated concentrations of Glabridin and PD98059 alone and in combination followed by staining with fluorescein‐conjugated Annexin‐V/PI, and apoptotic and necrotic cells were determined by flow cytometry. (B and C) ERK inhibition reversed Glabridin mediated inhibition of the spheroid formation in HH and H9 cells.

**Supplementary Figure S5.** (A) Heat map with hierarchical clustering of the significantly altered metabolites identified in samples of different experimental groups (control, Glabridin 80 μM, PD10μM and PD10μM+ Glabridin 80 μM), in CTCL (*p*‐value <0.05) (*n* = 3). Red represents high expression and green represents low expression with samples in column and row representing metabolites. Functional analysis of the significant features in Glabridin and Glabridin + ERK inhibitor treated CTCL cells (H9) using MetaboAnalyst 6.0 (https://www.metaboanalyst.ca/). (B) Quantitative enrichment analysis (QEA) overview representing the top 25 metabolic pathways. Within a particular metabolic pathway, enrichment ratio is calculated as the number of observed hits/expected hits. (C) Metabolome view of the important metabolic pathways. The pathway impact values (x‐axis) represent the influencing factor of topological analysis, and the –log(*p*) (y‐axis) represents the *p*‐value of the pathway enrichment analysis. Each circle represents a pathway, and the colour and size of each circle are based on the *p*‐value of the pathway enrichment analysis and pathways impact values from the pathway topology analysis, respectively.

**Supplementary Figure S6.** Quantitative assessment of the significant features. H9 cells were divided into four groups as control, Glabridin 80 μM, PD98059 10 μM and Glabridin 80 μM + PD98059 10 μM and treated with the indicated concentration of Glabridin and PD98059 alone and in combination for 24 h followed by metabolomics analysis as described in Materials and Methods. Next, the data were analysed, and all the box plots were generated using the MetaboAnalyst 6.0 (https://www.metaboanalyst.ca/). (A–F) Box plot (Box–Whisker plot) of the normalized concentration of important features identified by ANOVA plot with a *p*‐value <0.05 followed by Fisher's least significant difference method (Fisher's LSD) post hoc analyses. The Y‐axis of the box plot represents the normalized level of the original metabolite's concentration calculated as mean&amp;#x02009;±&amp;#x02009;SD.

**Supplementary Figure S7.** Glabridin targets signalling pathways associated with metabolic reprogramming. HH and H9 cells were treated with the indicated concentrations of Glabridin followed by cell lysate preparation and immunoblotting. (A–P) The western blot analysis of p‐AMPK, AMPK, C‐MYC, p‐AKT, AKT and Notch, and their relative quantification results are presented as mean ± SD (*n* = 3). The intensity of the bands was normalized with the respective loading control and quantified using image lab software. **p < 0.01, ***p < 0.001 and ****p < 0.0001 represent the level of significance between treatment groups relative to control group.

**Supplementary Table S1.** Significant features in the CTCL cells treated with Glabridin, and ERK inhibitor alone and in combination (One‐way ANOVA followed by the Fisher's least significant difference (LSD) test).

**Supplementary Table S2.** Metabolome view of the important metabolic pathways based on the significant features identified in Glabridin treated CTCL cells (H9) using the MetaboAnalyst 6.0 (https://www.metaboanalyst.ca/). The table displays eight columns including the metabolic pathway, match status, the *p*‐value, the −log10 (*p*) value, the Holm *p*‐value, false discovery rate (FDR) and the impact value.

**Supplementary Table S3.** Metabolome view of the important metabolic pathways based on the significant features identified between Glabridin versus Glabridin + ERK inhibitor treated CTCL cells (H9) using the MetaboAnalyst 6.0 (https://www.metaboanalyst.ca/). The table displays eight columns including the metabolic pathway, match status, the *p*‐value, the −log10 (*p*)‐value, the Holm *p*‐value, false discovery rate (FDR) and the impact value.

**Supplementary Table S4.** List of antibodies used.

## Data Availability

The data that support the findings of this study are available from the corresponding author upon reasonable request.
