# Supplementary figures and images for: Metabolomics analyses reveal the crucial role of ERK in regulating metabolic pathways associated with the proliferation of human cutaneous T‐cell lymphoma cells treated with Glabridin
Source: Cell Prolif. 2024 Jun 30;57(9):e13701. doi: 10.1111/cpr.13701 (PMC11503255; doi:10.1111/cpr.13701)

## Slide 1
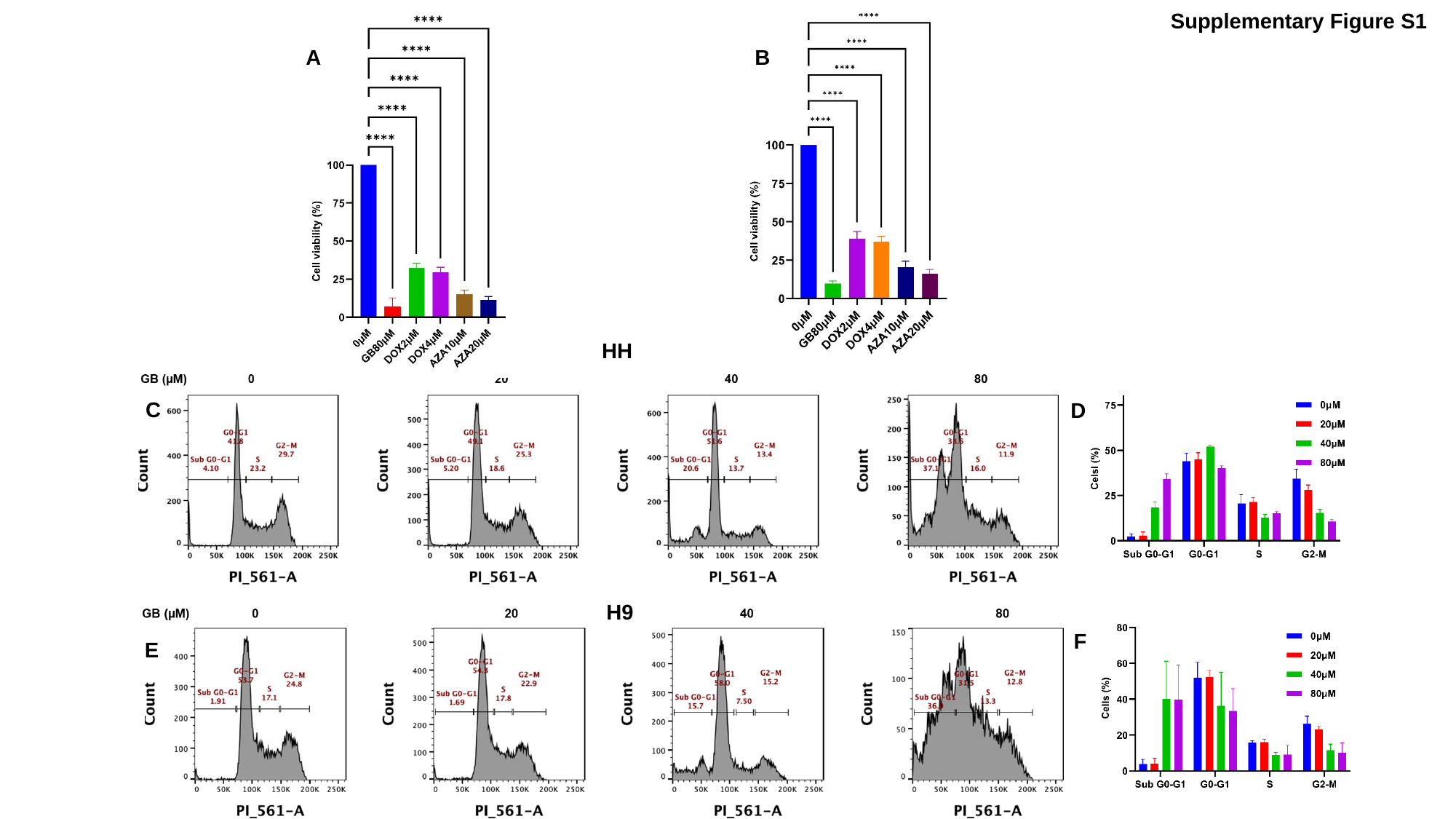

B
A
Supplementary Figure S1
HH
C
D
H9
E
F

Supplement: Supplementary file 1 — Supplementary Figure S1. Effect of Glabridin, doxorubicin (DOX) and azacytidine (AZA) on cell viability. (A) HH and (B) H9 cells were treated with the indicated concentration of GB, DOX and AZA for 24 h. CCK‐8 was used to determine the cell viability and data were presented as mean ± SD (n = 6). Effect of Glabridin on cell cycle distribution. (C and D) HH and (E and F) H9 cells were treated with different concentrations (0 μM, 20 μM, 40 μM, 80 μM), and the cell cycle distribution percentage was analysed by flow cytometry. Glabridin treatments induce a mark increase in the percentage of cells at the subG0‐G1 and G0‐G1 phase as compared to the control group in both HH (A and B) and H9 (C and D) cells. Results are presented as mean ± SD (n = 3). [file CPR-57-e13701-s003.pptx]

## Slide 1
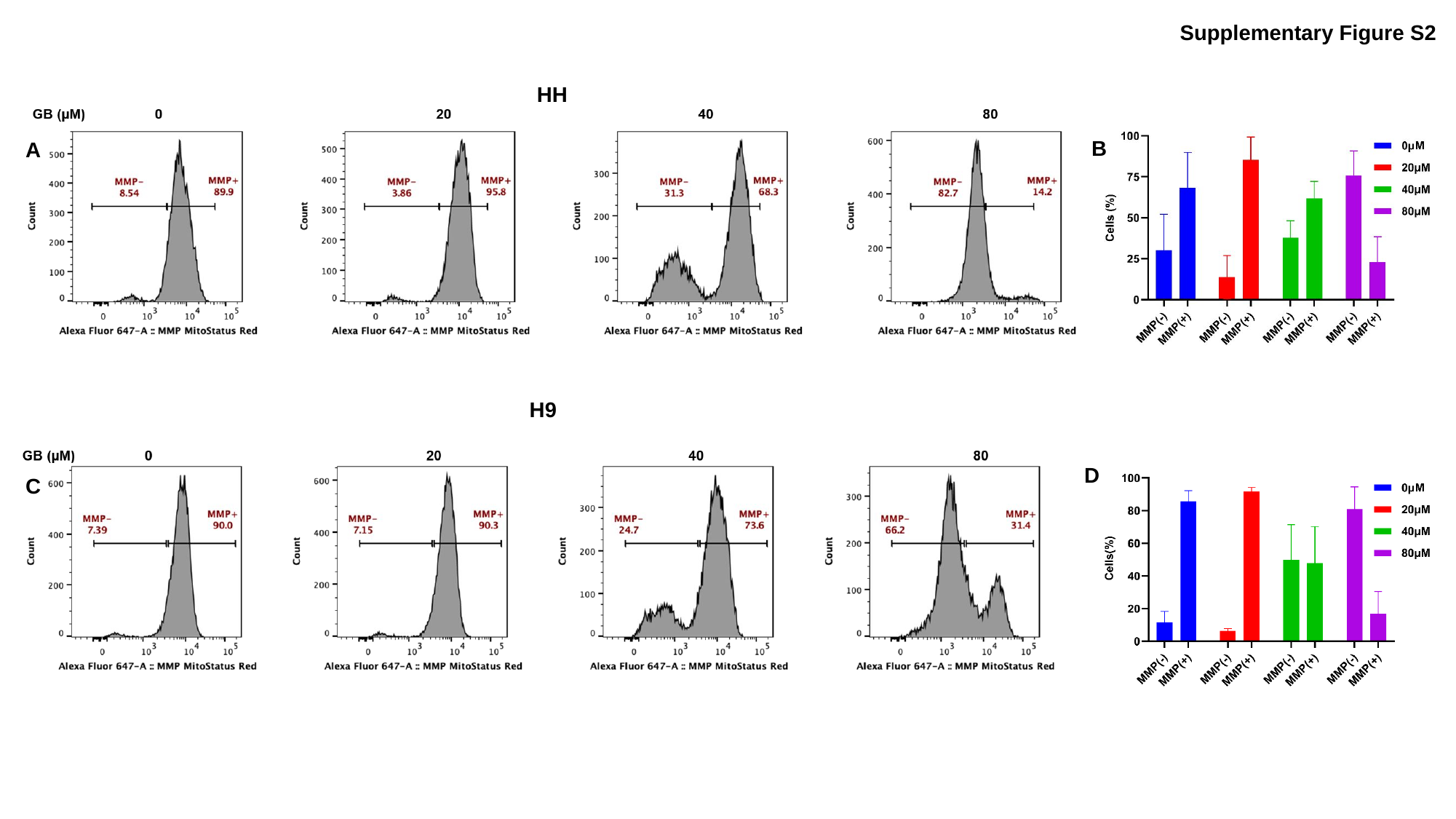

Supplementary Figure S2
HH
A
B
H9
C
D

Supplement: Supplementary file 2 — Supplementary Figure S2. Effect of Glabridin on the mitochondrial membrane potential. HH and H9 cells were treated with different concentrations (0 μM, 20 μM, 40 μM, 80 μM), and mitochondrial membrane potential was analysed by flow cytometry. Glabridin treatment induces a marked increase in the loss of mitochondrial membrane potential as compared to the control group in both HH (A and B) and H9 (C and D) cells. Results are presented as mean ± SD (n = 3). [file CPR-57-e13701-s008.pptx]

## Slide 1
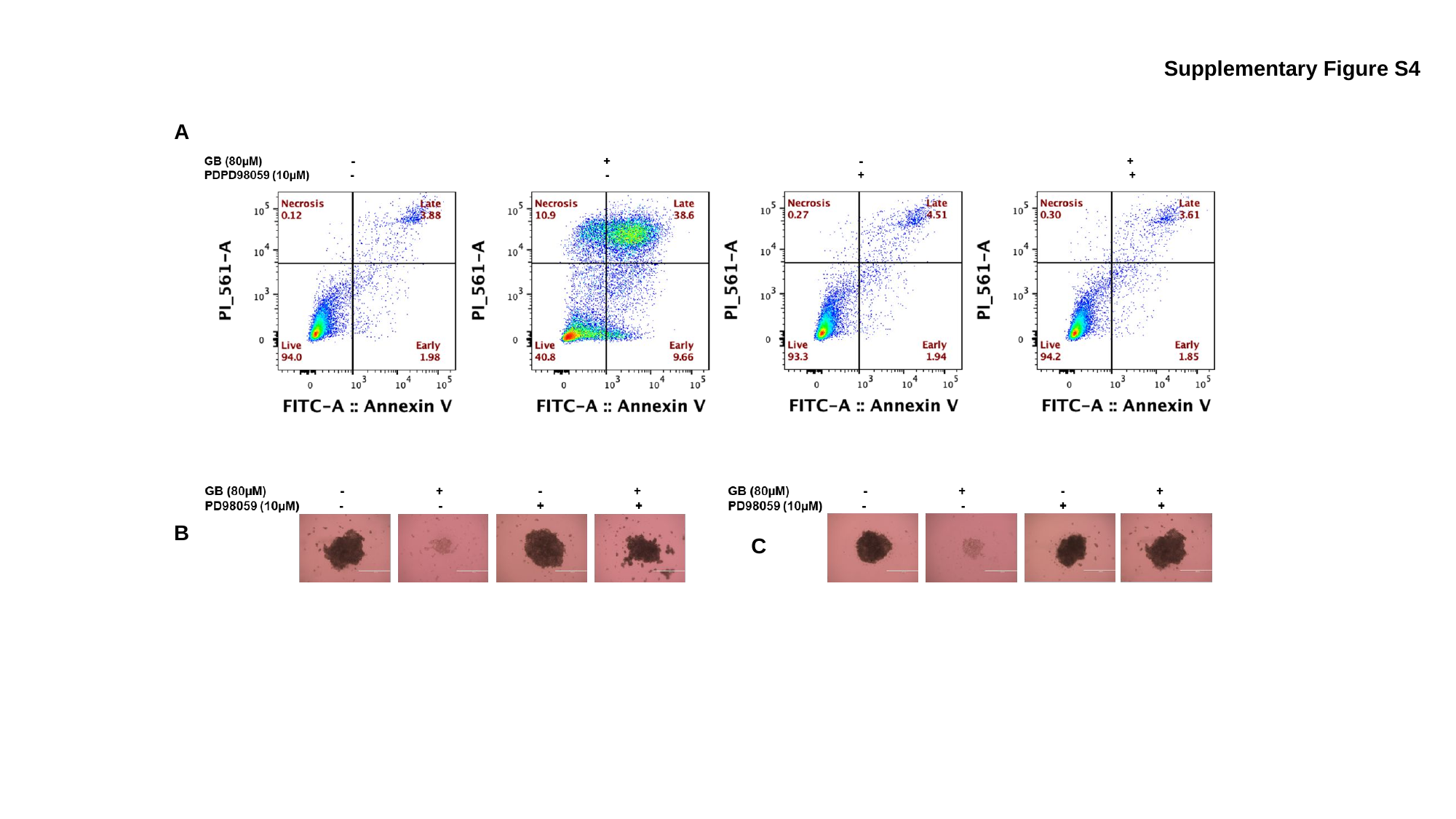

Supplementary Figure S4
A
B
C

Supplement: Supplementary file 4 — Supplementary Figure S4. (A) The data of H9 cells treated with the indicated concentrations of Glabridin and PD98059 alone and in combination followed by staining with fluorescein‐conjugated Annexin‐V/PI, and apoptotic and necrotic cells were determined by flow cytometry. (B and C) ERK inhibition reversed Glabridin mediated inhibition of the spheroid formation in HH and H9 cells. [file CPR-57-e13701-s006.pptx]
