# Supplementary material for: Metabolomics analyses reveal the crucial role of ERK in regulating metabolic pathways associated with the proliferation of human cutaneous T‐cell lymphoma cells treated with Glabridin
Source: Cell Prolif. 2024 Jun 30;57(9):e13701. doi: 10.1111/cpr.13701 (PMC11503255; doi:10.1111/cpr.13701)
Supplement: Supplementary file 3 — Supplementary Figure S3. Effect of Z‐Vad‐FMK on Glabridin induced apoptosis. HH and H9 cells were treated with the indicated concentration of Z‐VAD‐FMK and Glabridin (GB) alone in combination and then lysates were prepared, and immunoblotting was performed. (A–H) Western blot analysis of PARP, cleaved PARP and p‐H2AX and their relative quantification results are presented as mean ± SD (n = 3). (I–N) HH and H9 cells were treated with the indicated concentration of Glabridin in the presence and absence of 3‐MA and cell lysates were prepared followed by expression analysis and quantification of caspase‐3 and cleaved caspase‐3. The intensity of the bands was normalized with the respective loading control and quantified using image lab software. *p < 0.05 and **p < 0.01 represent the level of significance between treatment groups relative to control (positive and negative) groups. [file CPR-57-e13701-s007.pptx]

## Slide 1
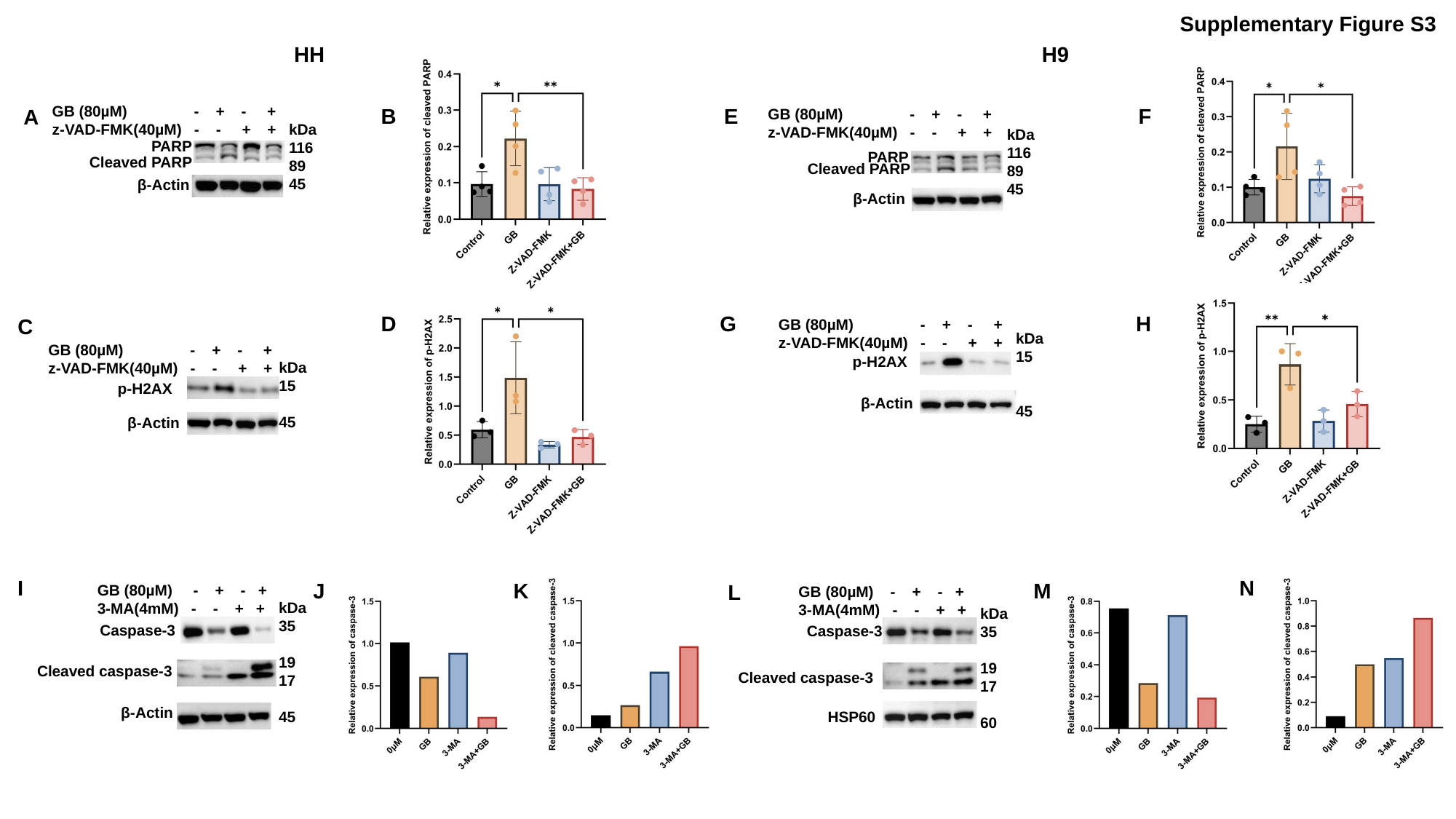

Supplementary Figure S3
H9
HH
GB (80µM) - + - +
z-VAD-FMK(40µM) - - + +
kDa
116
89
45
PARP
β-Actin
Cleaved PARP
B
E
F
A
GB (80µM) - + - +
z-VAD-FMK(40µM) - - + +
kDa
116
89
45
PARP
β-Actin
Cleaved PARP
D
H
G
C
GB (80µM) - + - +
z-VAD-FMK(40µM) - - + +
kDa
15
45
p-H2AX
β-Actin
GB (80µM) - + - +
z-VAD-FMK(40µM) - - + +
kDa
15
45
p-H2AX
β-Actin
I
N
J
M
K
L
GB (80µM) - + - +
3-MA(4mM) - - + +
kDa
35
19
17
45
Caspase-3
Cleaved caspase-3
β-Actin
GB (80µM) - + - +
3-MA(4mM) - - + +
kDa
35
19
17
60
Caspase-3
Cleaved caspase-3
HSP60
