# Supplementary material for: Metabolomics analyses reveal the crucial role of ERK in regulating metabolic pathways associated with the proliferation of human cutaneous T‐cell lymphoma cells treated with Glabridin
Source: Cell Prolif. 2024 Jun 30;57(9):e13701. doi: 10.1111/cpr.13701 (PMC11503255; doi:10.1111/cpr.13701)
Supplement: Supplementary file 5 — Supplementary Figure S5. (A) Heat map with hierarchical clustering of the significantly altered metabolites identified in samples of different experimental groups (control, Glabridin 80 μM, PD10μM and PD10μM+ Glabridin 80 μM), in CTCL (p‐value <0.05) (n = 3). Red represents high expression and green represents low expression with samples in column and row representing metabolites. Functional analysis of the significant features in Glabridin and Glabridin + ERK inhibitor treated CTCL cells (H9) using MetaboAnalyst 6.0 (https://www.metaboanalyst.ca/). (B) Quantitative enrichment analysis (QEA) overview representing the top 25 metabolic pathways. Within a particular metabolic pathway, enrichment ratio is calculated as the number of observed hits/expected hits. (C) Metabolome view of the important metabolic pathways. The pathway impact values (x‐axis) represent the influencing factor of topological analysis, and the –log(p) (y‐axis) represents the p‐value of the pathway enrichment analysis. Each circle represents a pathway, and the colour and size of each circle are based on the p‐value of the pathway enrichment analysis and pathways impact values from the pathway topology analysis, respectively. [file CPR-57-e13701-s002.pptx]

## Slide 1
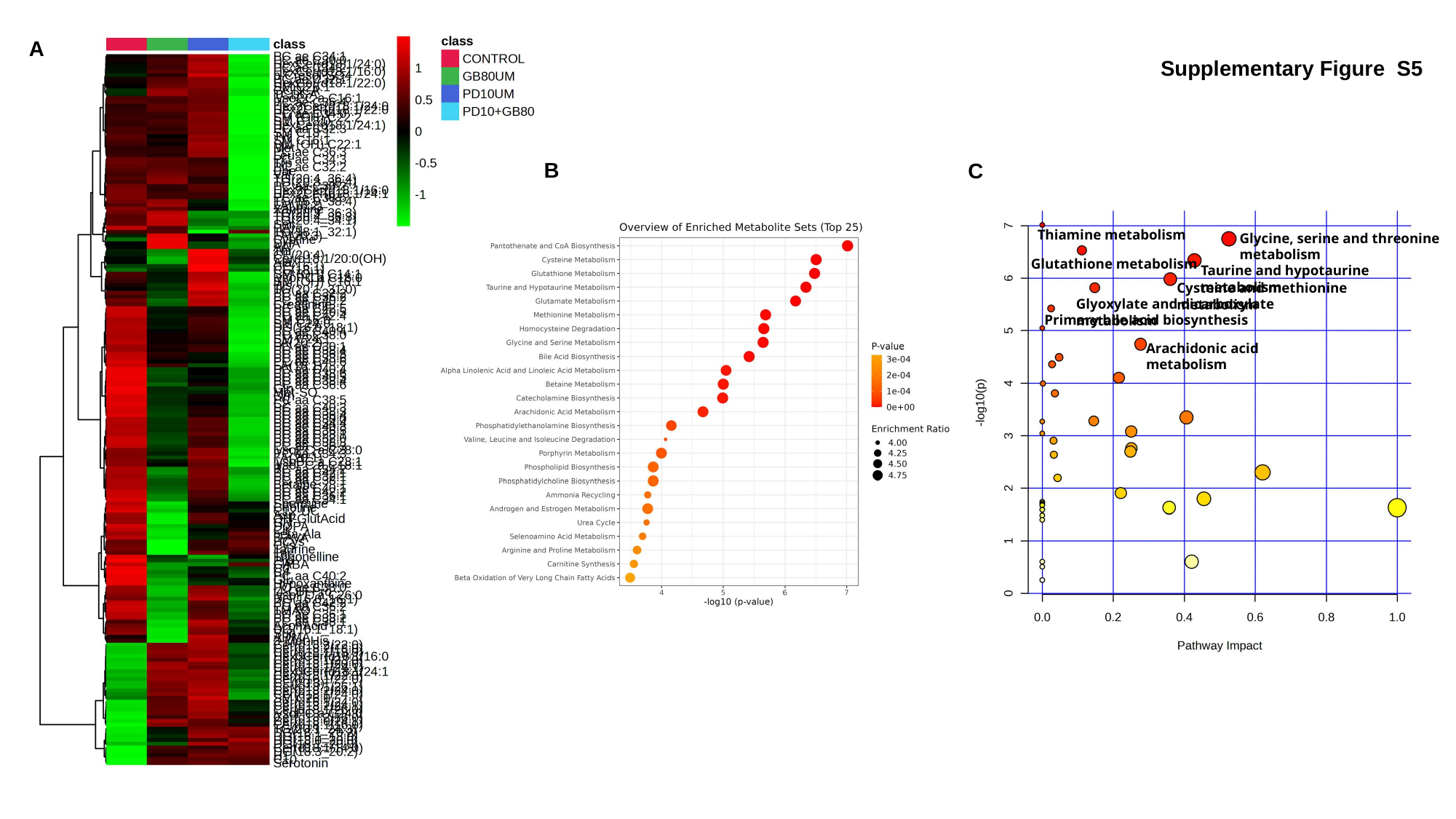

A
Supplementary Figure S5
B
C
Thiamine metabolism
Glycine, serine and threonine metabolism
Glutathione metabolism
Taurine and hypotaurine metabolism
Cysteine and methionine metabolism
Glyoxylate and dicarboxylate metabolism
Primary bile acid biosynthesis
Arachidonic acid metabolism
