# Supplementary material for: Metabolomics analyses reveal the crucial role of ERK in regulating metabolic pathways associated with the proliferation of human cutaneous T‐cell lymphoma cells treated with Glabridin
Source: Cell Prolif. 2024 Jun 30;57(9):e13701. doi: 10.1111/cpr.13701 (PMC11503255; doi:10.1111/cpr.13701)
Supplement: Supplementary file 6 — Supplementary Figure S6. Quantitative assessment of the significant features. H9 cells were divided into four groups as control, Glabridin 80 μM, PD98059 10 μM and Glabridin 80 μM + PD98059 10 μM and treated with the indicated concentration of Glabridin and PD98059 alone and in combination for 24 h followed by metabolomics analysis as described in Materials and Methods. Next, the data were analysed, and all the box plots were generated using the MetaboAnalyst 6.0 (https://www.metaboanalyst.ca/). (A–F) Box plot (Box–Whisker plot) of the normalized concentration of important features identified by ANOVA plot with a p‐value <0.05 followed by Fisher's least significant difference method (Fisher's LSD) post hoc analyses. The Y‐axis of the box plot represents the normalized level of the original metabolite's concentration calculated as mean&#x02009;±&#x02009;SD. [file CPR-57-e13701-s009.pptx]

## Slide 1
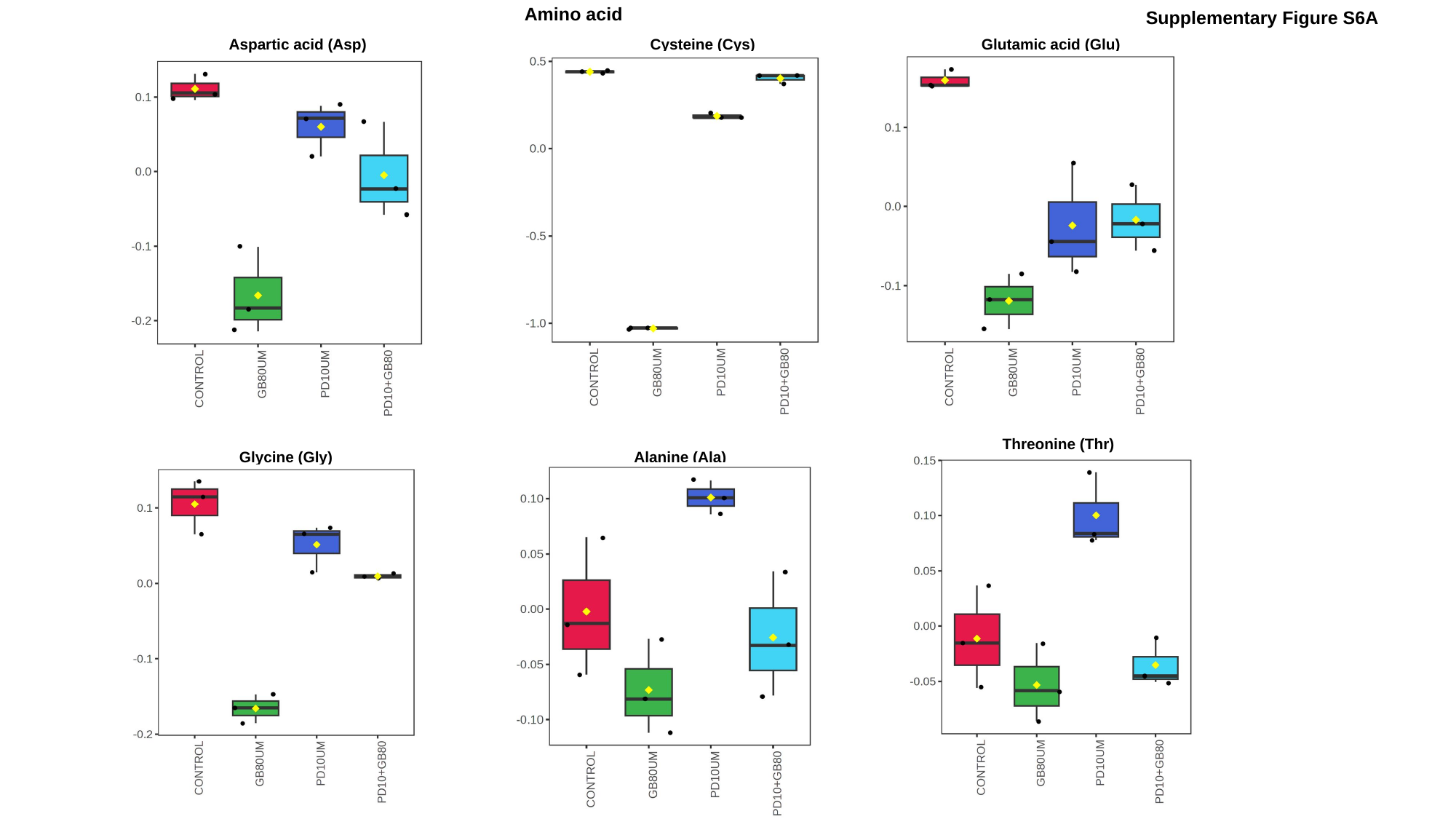

Amino acid
Aspartic acid (Asp)
Cysteine (Cys)
Glutamic acid (Glu)
Threonine (Thr)
Glycine (Gly)
Alanine (Ala)
Supplementary Figure S6A

## Slide 2
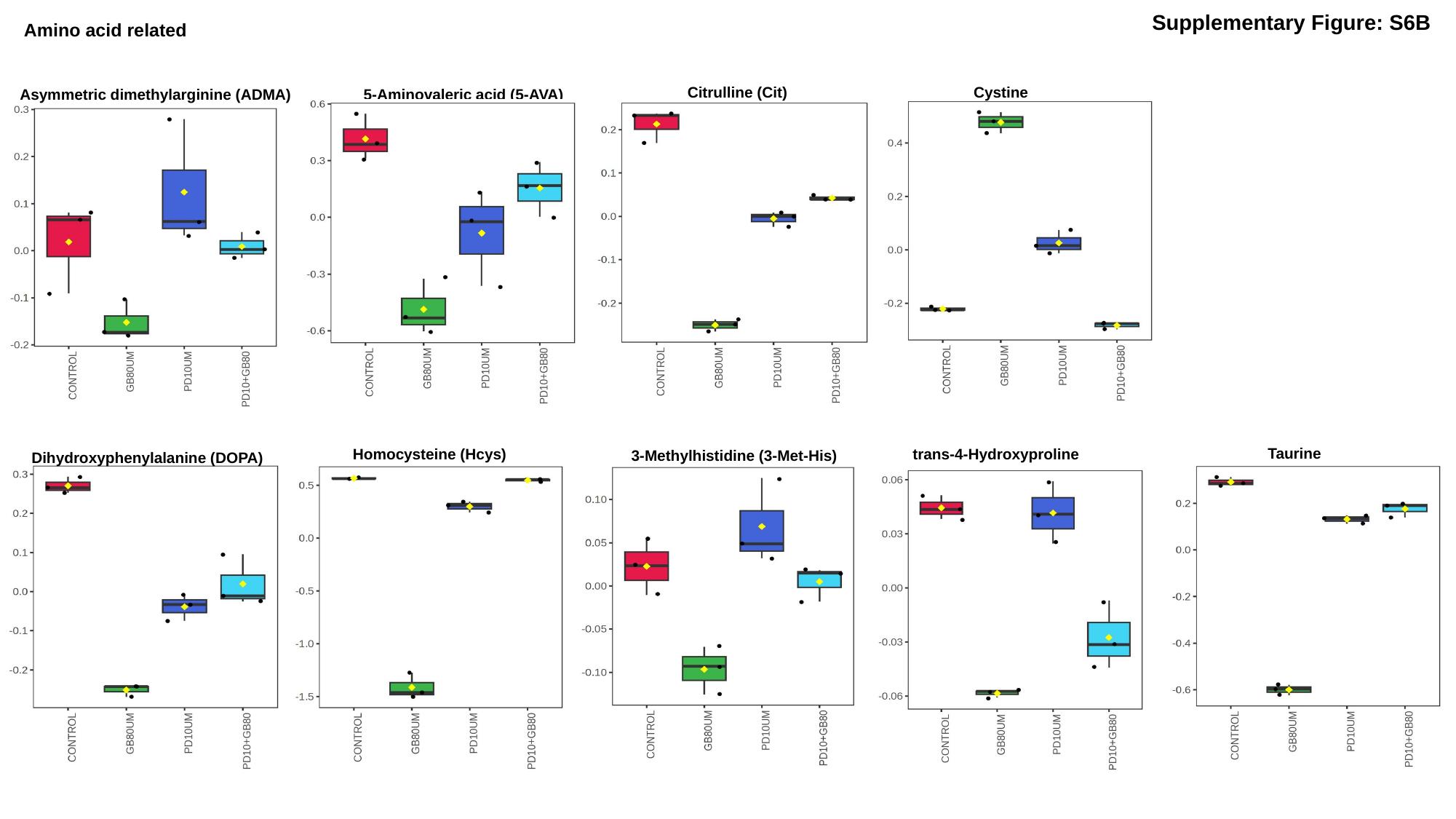

Supplementary Figure: S6B
Amino acid related
Citrulline (Cit)
Cystine
Asymmetric dimethylarginine (ADMA)
5-Aminovaleric acid (5-AVA)
Taurine
Homocysteine (Hcys)
trans-4-Hydroxyproline
3-Methylhistidine (3-Met-His)
Dihydroxyphenylalanine (DOPA)

## Slide 3
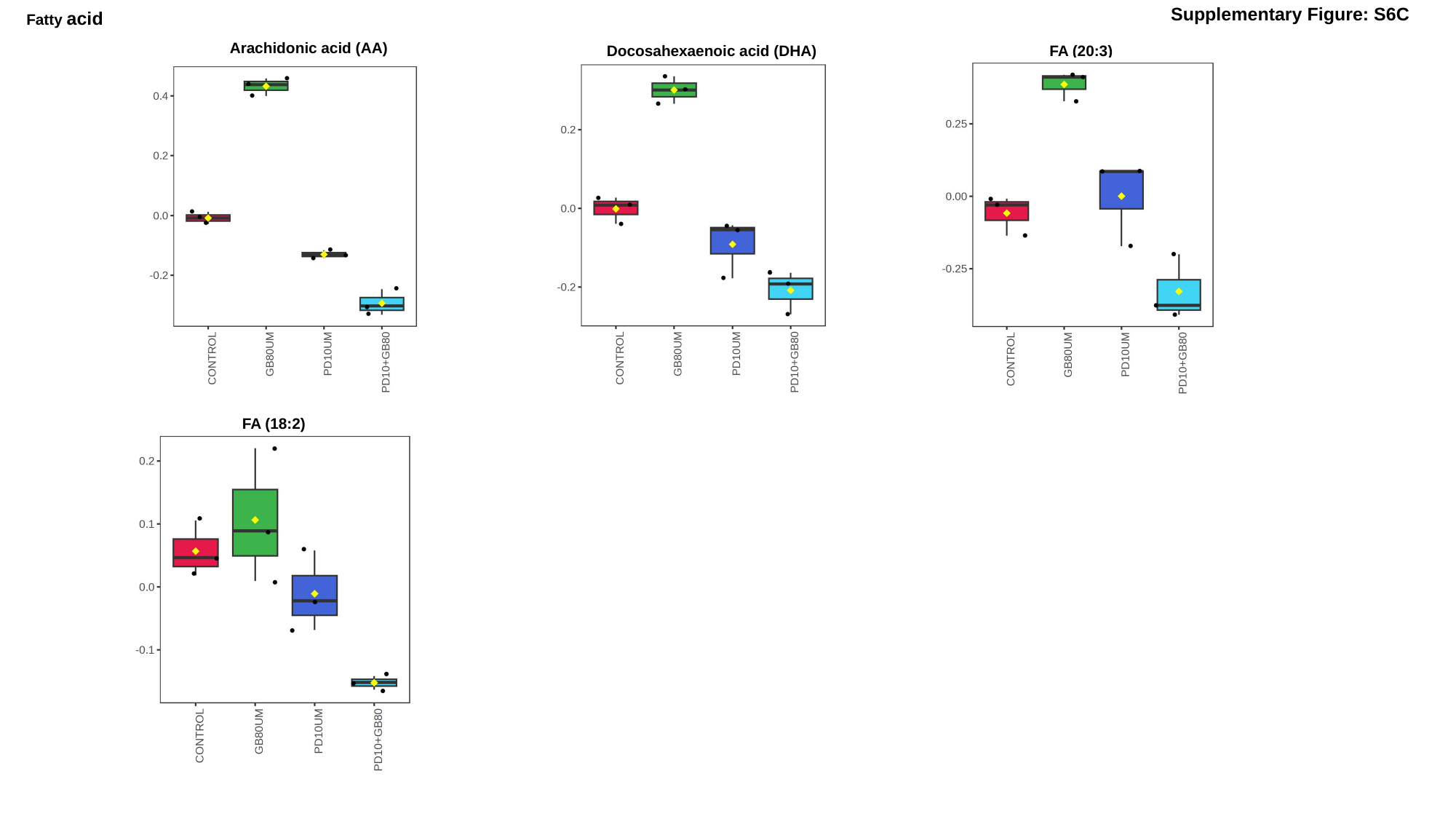

Supplementary Figure: S6C
Fatty acid
Arachidonic acid (AA)
Docosahexaenoic acid (DHA)
FA (20:3)
FA (18:2)

## Slide 4
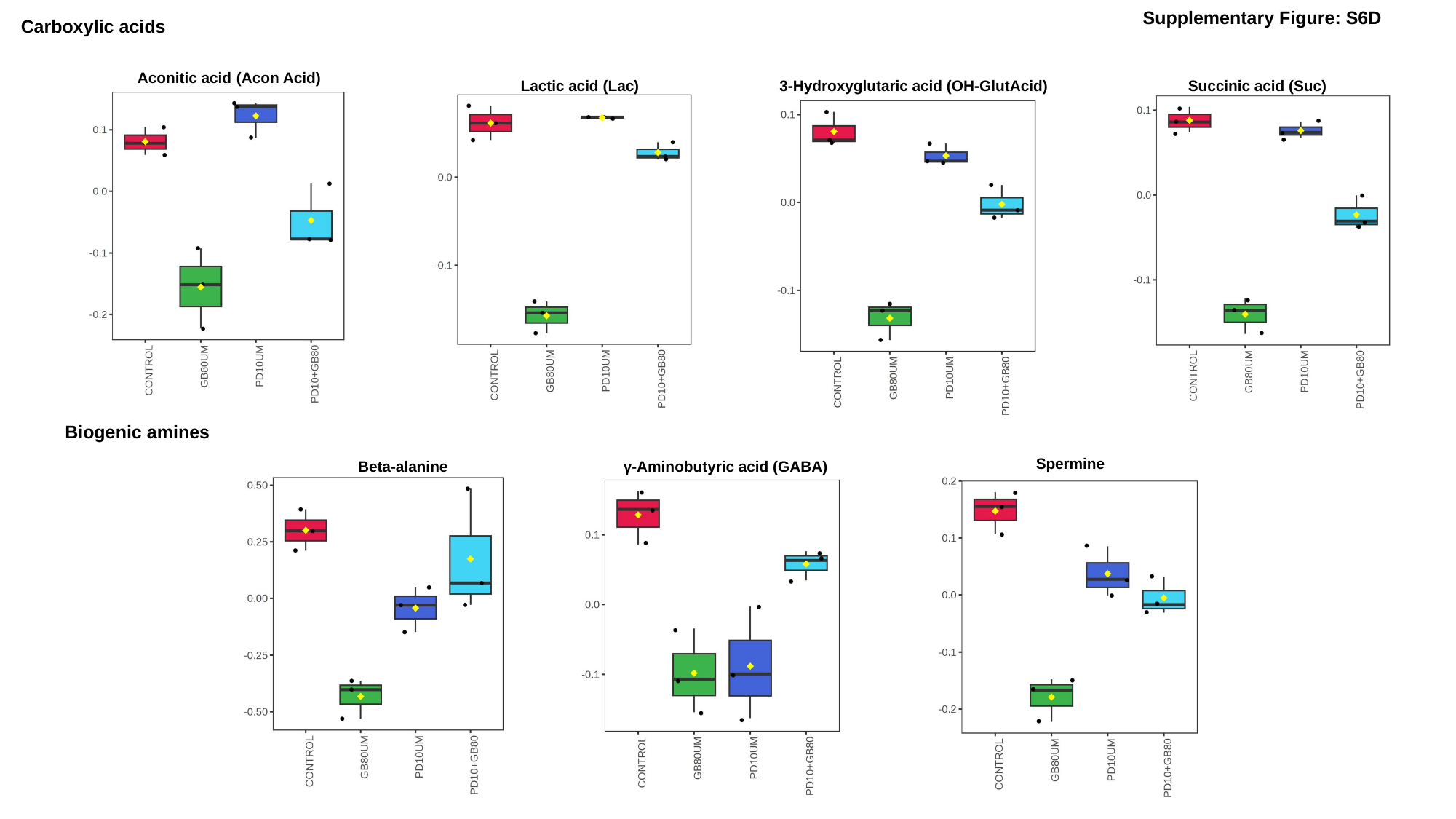

Supplementary Figure: S6D
Carboxylic acids
(Acon Acid)
Aconitic acid
Lactic acid (Lac)
3-Hydroxyglutaric acid (OH-GlutAcid)
Succinic acid (Suc)
Biogenic amines
Spermine
Beta-alanine
γ-Aminobutyric acid (GABA)

## Slide 5
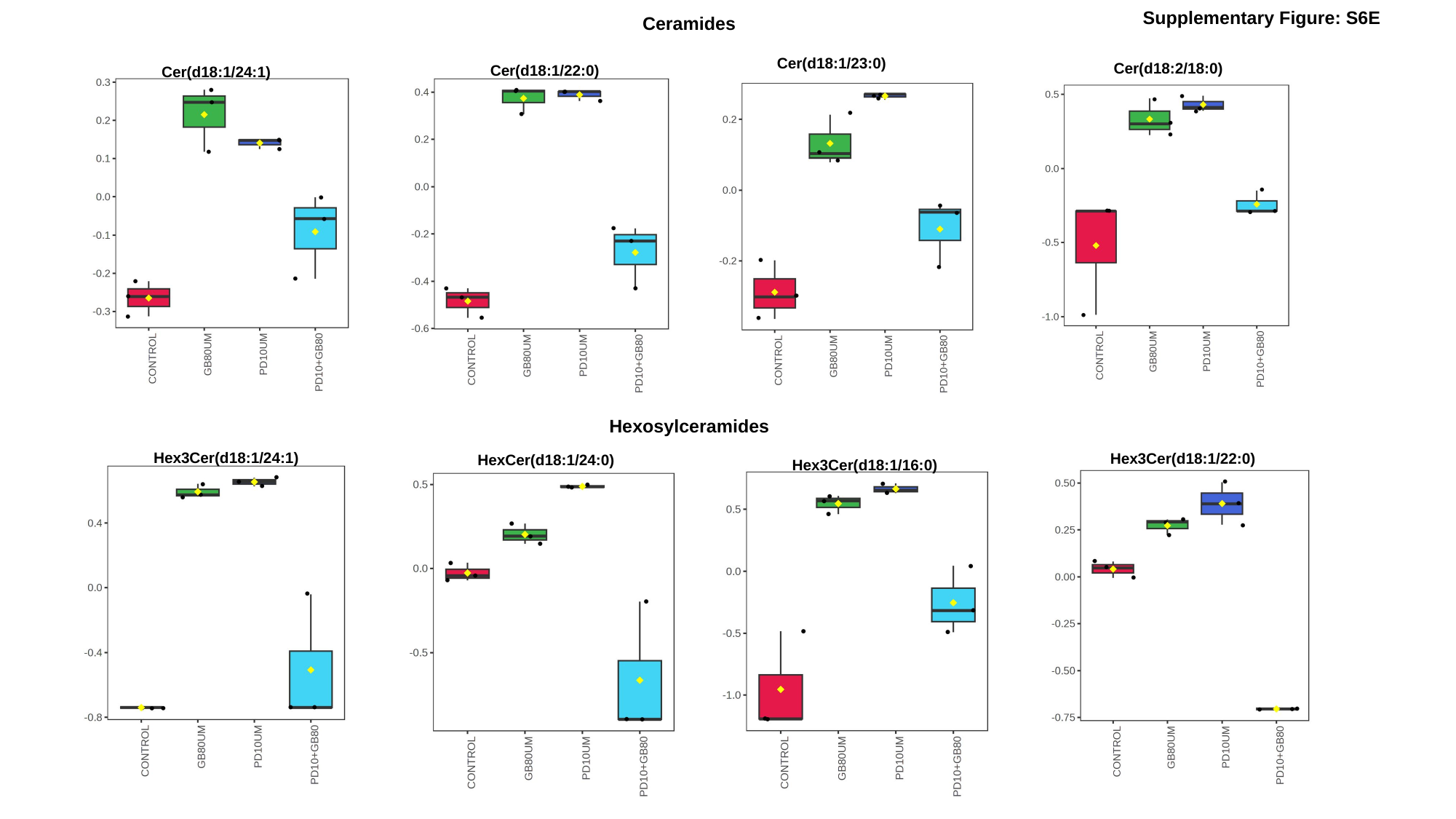

Supplementary Figure: S6E
Ceramides
Cer(d18:1/23:0)
Cer(d18:2/18:0)
Cer(d18:1/22:0)
Cer(d18:1/24:1)
Hexosylceramides
Hex3Cer(d18:1/24:1)
Hex3Cer(d18:1/22:0)
Hex3Cer(d18:1/16:0)
HexCer(d18:1/24:0)

## Slide 6
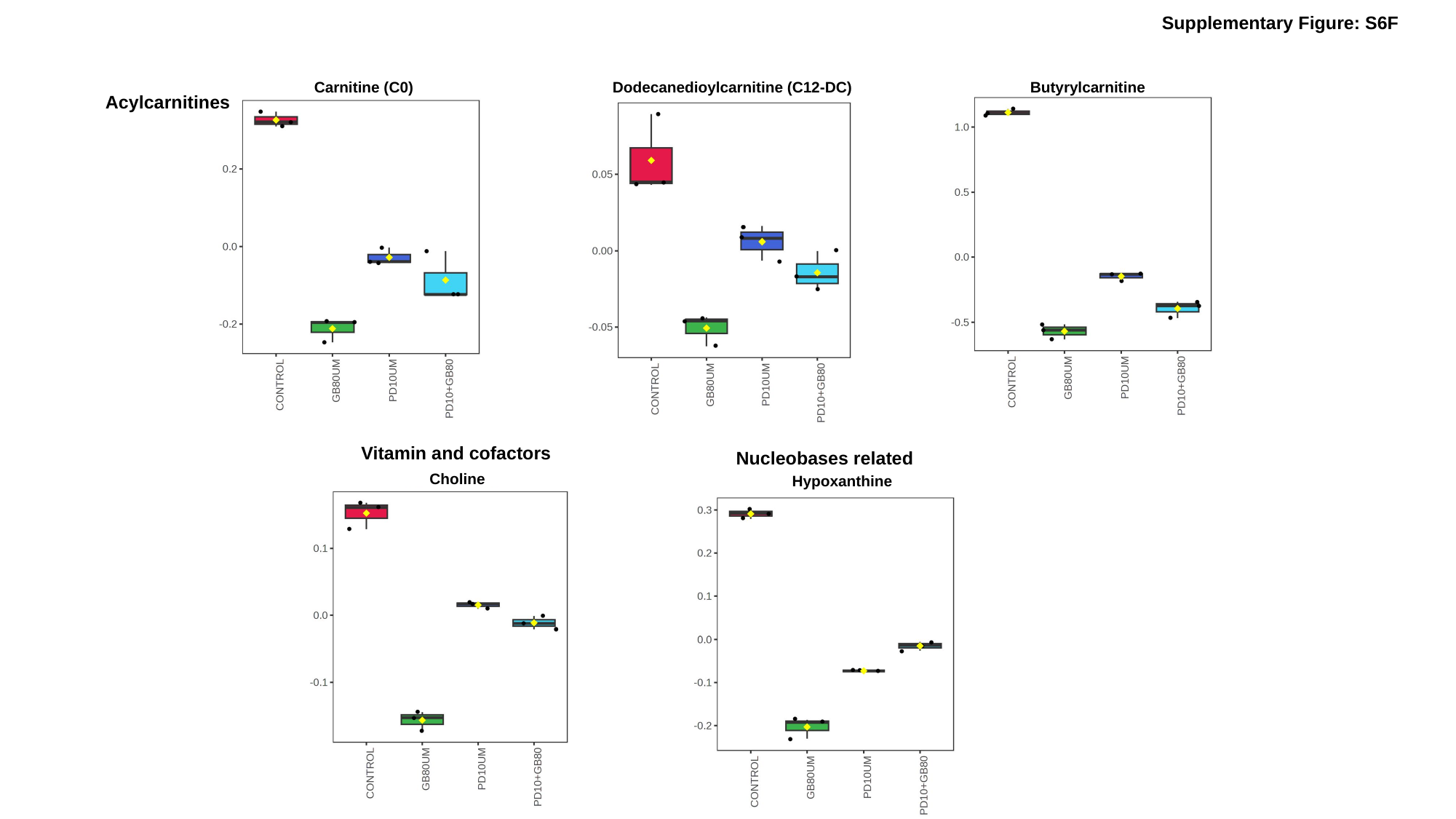

Supplementary Figure: S6F
Carnitine (C0)
Dodecanedioylcarnitine (C12-DC)
Butyrylcarnitine
Acylcarnitines
Vitamin and cofactors
Choline
Nucleobases related
Hypoxanthine
