# Supplementary material for: Metabolomics analyses reveal the crucial role of ERK in regulating metabolic pathways associated with the proliferation of human cutaneous T‐cell lymphoma cells treated with Glabridin
Source: Cell Prolif. 2024 Jun 30;57(9):e13701. doi: 10.1111/cpr.13701 (PMC11503255; doi:10.1111/cpr.13701)
Supplement: Supplementary file 7 — Supplementary Figure S7. Glabridin targets signalling pathways associated with metabolic reprogramming. HH and H9 cells were treated with the indicated concentrations of Glabridin followed by cell lysate preparation and immunoblotting. (A–P) The western blot analysis of p‐AMPK, AMPK, C‐MYC, p‐AKT, AKT and Notch, and their relative quantification results are presented as mean ± SD (n = 3). The intensity of the bands was normalized with the respective loading control and quantified using image lab software. **p < 0.01, ***p < 0.001 and ****p < 0.0001 represent the level of significance between treatment groups relative to control group. [file CPR-57-e13701-s004.pptx]

## Slide 1
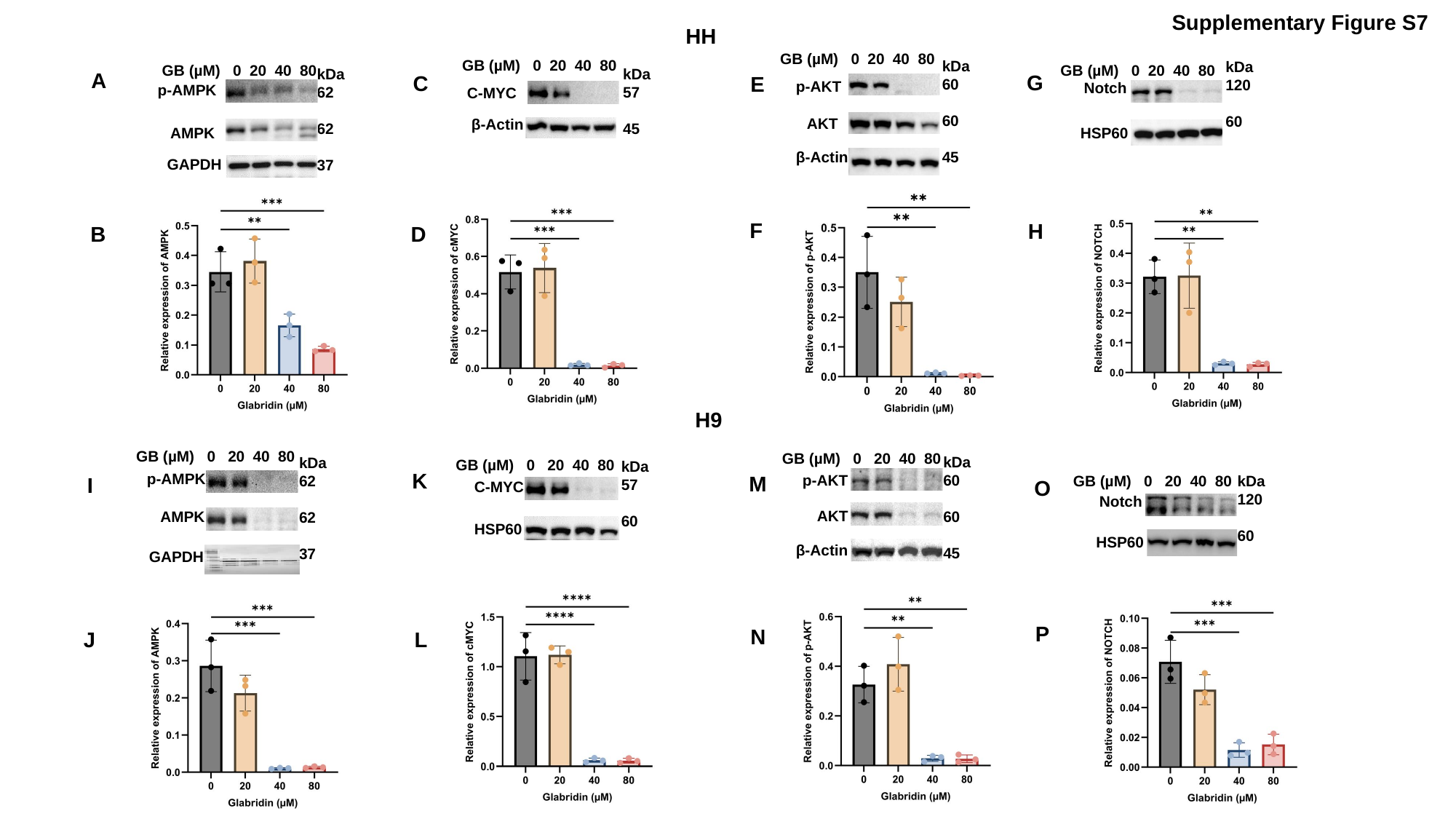

Supplementary Figure S7
HH
GB (µM) 0 20 40 80
kDa
60
60
45
p-AKT
AKT
β-Actin
GB (µM) 0 20 40 80
kDa
60
60
45
p-AKT
AKT
β-Actin
E
F
M
N
GB (µM) 0 20 40 80
kDa
57
45
C-MYC
β-Actin
GB (µM) 0 20 40 80
kDa
57
60
C-MYC
HSP60
C
D
K
L
kDa
120
60
GB (µM) 0 20 40 80
Notch
HSP60
GB (µM) 0 20 40 80
kDa
120
60
Notch
HSP60
G
H
O
P
GB (µM) 0 20 40 80
kDa
62
62
37
p-AMPK
AMPK
GAPDH
GB (µM) 0 20 40 80
kDa
62
62
37
p-AMPK
AMPK
GAPDH
A
B
I
J
D
H9
