# Supplementary material for: Metabolomics analyses reveal the crucial role of ERK in regulating metabolic pathways associated with the proliferation of human cutaneous T‐cell lymphoma cells treated with Glabridin
Source: Cell Prolif. 2024 Jun 30;57(9):e13701. doi: 10.1111/cpr.13701 (PMC11503255; doi:10.1111/cpr.13701)
Supplement: Supplementary file 8 — Supplementary Table S1. Significant features in the CTCL cells treated with Glabridin, and ERK inhibitor alone and in combination (One‐way ANOVA followed by the Fisher's least significant difference (LSD) test). [file CPR-57-e13701-s001.docx]

| **Features/Metabolite** | **f.value** | **p.value** | **FDR** |
| --- | --- | --- | --- |
| Cys | 5456.5 | 1.40E-13 | 3.43E-11 |
| Taurine | 888.84 | 1.97E-10 | 2.16E-08 |
| PC ae C40:4 | 825.26 | 2.65E-10 | 2.16E-08 |
| C4 | 737.4 | 4.14E-10 | 2.35E-08 |
| PC aa C42:4 | 696.96 | 5.19E-10 | 2.35E-08 |
| Hypoxanthine | 678.77 | 5.76E-10 | 2.35E-08 |
| HCys | 610.49 | 8.79E-10 | 2.74E-08 |
| Hex2Cer(d18:1/16:0) | 607.93 | 8.94E-10 | 2.74E-08 |
| PC ae C38:4 | 585.72 | 1.04E-09 | 2.82E-08 |
| PC aa C38:6 | 531.42 | 1.53E-09 | 3.74E-08 |
| C2 | 486.41 | 2.17E-09 | 4.83E-08 |
| PC aa C36:3 | 416.95 | 4.00E-09 | 8.17E-08 |
| Cystine | 378.51 | 5.88E-09 | 1.11E-07 |
| PC aa C38:3 | 350.54 | 7.97E-09 | 1.36E-07 |
| AA | 346.93 | 8.31E-09 | 1.36E-07 |
| PC aa C38:4 | 322.6 | 1.11E-08 | 1.70E-07 |
| PC ae C40:5 | 304.86 | 1.39E-08 | 2.00E-07 |
| PC aa C40:3 | 295.69 | 1.56E-08 | 2.05E-07 |
| PC aa C34:4 | 294.68 | 1.59E-08 | 2.05E-07 |
| PC aa C36:4 | 285.57 | 1.80E-08 | 2.10E-07 |
| PC ae C40:6 | 285.43 | 1.80E-08 | 2.10E-07 |
| PC aa C40:5 | 258.65 | 2.66E-08 | 2.96E-07 |
| Choline | 249 | 3.09E-08 | 3.29E-07 |
| Cit | 223.82 | 4.71E-08 | 4.80E-07 |
| PC aa C34:3 | 216.54 | 5.36E-08 | 5.26E-07 |
| Cer(d18:1/16:0) | 198.94 | 7.49E-08 | 7.03E-07 |
| PC aa C38:5 | 197.15 | 7.76E-08 | 7.03E-07 |
| PC aa C40:4 | 195.44 | 8.04E-08 | 7.03E-07 |
| PC ae C38:6 | 183.19 | 1.04E-07 | 8.76E-07 |
| PC ae C32:2 | 180.68 | 1.09E-07 | 8.94E-07 |
| PC ae C38:5 | 176.76 | 1.19E-07 | 9.43E-07 |
| HexCer(d18:1/22:0) | 172.8 | 1.30E-07 | 9.99E-07 |
| Lac | 164.39 | 1.59E-07 | 1.18E-06 |
| PC aa C36:5 | 159.6 | 1.78E-07 | 1.28E-06 |
| PC ae C32:1 | 153.85 | 2.06E-07 | 1.44E-06 |
| PC ae C30:1 | 145.95 | 2.53E-07 | 1.72E-06 |
| Hex2Cer(d18:1/22:0) | 136.66 | 3.28E-07 | 2.17E-06 |
| Serotonin | 124.38 | 4.74E-07 | 3.05E-06 |
| PC ae C38:3 | 121.45 | 5.20E-07 | 3.27E-06 |
| C10 | 119.71 | 5.50E-07 | 3.37E-06 |
| SM C24:1 | 116.63 | 6.09E-07 | 3.59E-06 |
| PC aa C36:1 | 116.32 | 6.15E-07 | 3.59E-06 |
| Suc | 114.59 | 6.52E-07 | 3.72E-06 |
| C0 | 105.83 | 8.89E-07 | 4.95E-06 |
| PC aa C42:1 | 103.4 | 9.73E-07 | 5.30E-06 |
| PC ae C36:4 | 95.451 | 1.33E-06 | 7.07E-06 |
| Cer(d18:1/22:0) | 93.648 | 1.43E-06 | 7.34E-06 |
| Cer(d18:1/24:0) | 93.197 | 1.46E-06 | 7.34E-06 |
| PC ae C30:0 | 93.032 | 1.47E-06 | 7.34E-06 |
| PC aa C32:0 | 91.355 | 1.57E-06 | 7.71E-06 |
| Hex2Cer(d18:1/24:1) | 90.58 | 1.63E-06 | 7.82E-06 |
| DOPA | 89.941 | 1.67E-06 | 7.88E-06 |
| Hex2Cer(d18:1/24:0) | 86.738 | 1.92E-06 | 8.90E-06 |
| OH-GlutAcid | 76.897 | 3.06E-06 | 1.39E-05 |
| PC aa C36:2 | 68.568 | 4.76E-06 | 2.12E-05 |
| PC aa C34:1 | 65.962 | 5.52E-06 | 2.41E-05 |
| SM C18:1 | 65.735 | 5.60E-06 | 2.41E-05 |
| PC aa C30:0 | 63.596 | 6.35E-06 | 2.68E-05 |
| PC ae C34:2 | 62.639 | 6.73E-06 | 2.79E-05 |
| PC aa C40:2 | 61.399 | 7.26E-06 | 2.95E-05 |
| Gly | 61.194 | 7.36E-06 | 2.95E-05 |
| HexCer(d18:1/16:0) | 57.029 | 9.62E-06 | 3.80E-05 |
| PC aa C34:2 | 55.327 | 1.08E-05 | 4.20E-05 |
| DHA | 52.388 | 1.33E-05 | 5.08E-05 |
| Ser | 51.679 | 1.40E-05 | 5.27E-05 |
| Cer(d18:2/24:0) | 49.495 | 1.65E-05 | 6.11E-05 |
| Creatinine | 47.832 | 1.87E-05 | 6.85E-05 |
| SM C16:1 | 46.315 | 2.11E-05 | 7.62E-05 |
| lysoPC a C18:1 | 45.955 | 2.18E-05 | 7.73E-05 |
| t4-OH-Pro | 44.68 | 2.42E-05 | 8.47E-05 |
| TG(18:1_32:1) | 44.206 | 2.52E-05 | 8.69E-05 |
| PC ae C34:1 | 43.94 | 2.58E-05 | 8.77E-05 |
| PC aa C42:5 | 43.756 | 2.62E-05 | 8.78E-05 |
| PC aa C36:6 | 42.051 | 3.04E-05 | 0.000101 |
| SM C24:0 | 40.81 | 3.40E-05 | 0.000111 |
| Hex3Cer(d18:1/24:1) | 38.304 | 4.30E-05 | 0.000139 |
| Cer(d18:2/24:1) | 37.664 | 4.58E-05 | 0.000146 |
| TG(20:3_36:4) | 37.169 | 4.81E-05 | 0.00015 |
| FA(20:2) | 37.002 | 4.89E-05 | 0.00015 |
| Spermine | 36.982 | 4.90E-05 | 0.00015 |
| PC aa C32:1 | 35.279 | 5.83E-05 | 0.000176 |
| H1 | 35.117 | 5.93E-05 | 0.000177 |
| PC ae C36:2 | 34.489 | 6.34E-05 | 0.000187 |
| Cer(d18:1/18:0) | 34.245 | 6.51E-05 | 0.000188 |
| Betaine | 34.222 | 6.52E-05 | 0.000188 |
| Cer(d18:1/23:0) | 33.862 | 6.78E-05 | 0.000193 |
| PC ae C36:3 | 32.738 | 7.68E-05 | 0.000216 |
| Trp | 32.21 | 8.15E-05 | 0.000227 |
| PC aa C32:2 | 31.703 | 8.64E-05 | 0.000238 |
| Cer(d18:2/16:0) | 30.341 | 0.000101 | 0.000276 |
| SM (OH) C22:2 | 30.039 | 0.000105 | 0.000283 |
| Cer(d18:1/26:1) | 29.143 | 0.000117 | 0.000313 |
| PC ae C42:2 | 27.897 | 0.000138 | 0.000359 |
| PC ae C36:5 | 27.886 | 0.000138 | 0.000359 |
| Hex3Cer(d18:1/16:0) | 27.475 | 0.000145 | 0.000375 |
| SM C26:1 | 27.359 | 0.000148 | 0.000377 |
| p-Cresol-SO4 | 27.131 | 0.000152 | 0.000383 |
| PC aa C42:2 | 27.078 | 0.000153 | 0.000383 |
| Pro | 26.669 | 0.000162 | 0.0004 |
| Cer(d18:1/24:1) | 26.355 | 0.000169 | 0.000414 |
| PC ae C34:3 | 25.467 | 0.000191 | 0.000463 |
| TCDCA | 24.967 | 0.000205 | 0.00049 |
| Cer(d18:1/20:0) | 24.928 | 0.000206 | 0.00049 |
| FA(20:3) | 24.718 | 0.000212 | 0.000501 |
| Xanthine | 24.625 | 0.000215 | 0.000503 |
| C12-DC | 23.223 | 0.000265 | 0.000613 |
| PC aa C32:3 | 22.691 | 0.000288 | 0.000658 |
| AconAcid | 22.648 | 0.00029 | 0.000658 |
| PC aa C30:2 | 21.825 | 0.00033 | 0.000743 |
| TG(20:4_34:1) | 20.714 | 0.000397 | 0.000884 |
| Glu | 19.986 | 0.00045 | 0.000985 |
| PC aa C38:0 | 19.978 | 0.00045 | 0.000985 |
| CE(18:1) | 19.348 | 0.000503 | 0.001092 |
| Asp | 18.989 | 0.000537 | 0.001155 |
| HexCer(d18:1/24:0) | 17.011 | 0.000784 | 0.001669 |
| DG(18:3_20:2) | 16.711 | 0.000833 | 0.001758 |
| lysoPC a C16:0 | 15.599 | 0.001051 | 0.0022 |
| Cer(d16:1/22:0) | 15.451 | 0.001085 | 0.002252 |
| SM C16:0 | 15.275 | 0.001127 | 0.002319 |
| lysoPC a C28:0 | 15.239 | 0.001136 | 0.002319 |
| lysoPC a C26:0 | 14.827 | 0.001245 | 0.002521 |
| CE(20:3) | 14.603 | 0.00131 | 0.00263 |
| PC aa C28:1 | 14.544 | 0.001327 | 0.002644 |
| 5-AVA | 14.464 | 0.001352 | 0.002671 |
| CE(20:4) | 14.082 | 0.001477 | 0.002896 |
| PC ae C34:0 | 13.767 | 0.001592 | 0.003095 |
| Cer(d18:2/18:0) | 13.295 | 0.001784 | 0.003442 |
| SM C26:0 | 12.838 | 0.001999 | 0.003827 |
| Cer(d18:1/26:0) | 12.734 | 0.002053 | 0.003899 |
| GABA | 12.378 | 0.00225 | 0.004202 |
| 3-Met-His | 12.368 | 0.002256 | 0.004202 |
| beta-Ala | 12.335 | 0.002275 | 0.004202 |
| HexCer(d18:1/24:1) | 12.325 | 0.002281 | 0.004202 |
| PC ae C38:2 | 12.162 | 0.002381 | 0.004353 |
| DG(18:1_20:0) | 11.682 | 0.002708 | 0.004915 |
| Cer(d18:1/14:0) | 11.549 | 0.002809 | 0.00506 |
| SM (OH) C22:1 | 11.448 | 0.002888 | 0.005165 |
| PC ae C38:0 | 11.365 | 0.002956 | 0.005247 |
| DG(16:1_18:1) | 11.316 | 0.002997 | 0.005282 |
| Thr | 11.283 | 0.003025 | 0.005293 |
| SM C18:0 | 11.235 | 0.003065 | 0.005326 |
| TG(20:1_31:0) | 11.11 | 0.003175 | 0.005478 |
| DG(16:1_18:0) | 11.075 | 0.003207 | 0.005495 |
| Met-SO | 10.912 | 0.003361 | 0.005718 |
| PC aa C38:1 | 10.689 | 0.003584 | 0.006056 |
| GCDCA | 10.631 | 0.003646 | 0.006118 |
| lysoPC a C16:1 | 10.494 | 0.003796 | 0.006327 |
| Tyr | 10.449 | 0.003848 | 0.00637 |
| DG(18:1_20:2) | 10.116 | 0.004254 | 0.006995 |
| Cer(d18:0/24:1) | 10.022 | 0.004377 | 0.007131 |
| Gln | 10.009 | 0.004395 | 0.007131 |
| PC ae C36:1 | 9.8857 | 0.004566 | 0.00736 |
| CE(16:1) | 9.7934 | 0.004699 | 0.007525 |
| Met | 9.574 | 0.005036 | 0.008012 |
| TG(20:1_24:3) | 9.071 | 0.005929 | 0.009372 |
| lysoPC a C24:0 | 9.0198 | 0.006031 | 0.009471 |
| Cer(d18:2/22:0) | 8.6876 | 0.006746 | 0.010527 |
| FA(18:2) | 8.6436 | 0.006848 | 0.01059 |
| PC ae C40:3 | 8.6333 | 0.006872 | 0.01059 |
| TG(20:4_36:4) | 8.3983 | 0.007457 | 0.011418 |
| TMAO | 7.965 | 0.008706 | 0.013247 |
| FA(20:1) | 7.5942 | 0.009988 | 0.015105 |
| Cer(d16:1/24:0) | 7.0316 | 0.012419 | 0.018667 |
| Ala | 7.0037 | 0.012558 | 0.01876 |
| TG(16:0_38:4) | 6.8716 | 0.013241 | 0.019662 |
| DG(16:0_18:1) | 6.8188 | 0.013528 | 0.019966 |
| Val | 6.7668 | 0.013818 | 0.020272 |
| Trigonelline | 6.7179 | 0.014097 | 0.020558 |
| PC ae C40:1 | 6.3258 | 0.016613 | 0.024083 |
| Cer(d18:1/20:0(OH)) | 6.2154 | 0.01742 | 0.025105 |
| PC ae C38:1 | 6.1866 | 0.017639 | 0.025273 |
| SM (OH) C14:1 | 6.0839 | 0.018447 | 0.026276 |
| SM (OH) C16:1 | 6.0025 | 0.01912 | 0.027078 |
| TG(20:4_36:3) | 5.6031 | 0.022906 | 0.032252 |
| DG(16:0_16:1) | 5.4849 | 0.024202 | 0.033883 |
| PC ae C40:2 | 5.39 | 0.02531 | 0.035232 |
| TG(20:3_36:3) | 5.2795 | 0.026679 | 0.036925 |
| Orn | 5.268 | 0.026827 | 0.036925 |
| ADMA | 5.2414 | 0.027173 | 0.037192 |
| FA(18:1) | 5.1563 | 0.028316 | 0.038541 |
| Leu | 5.1323 | 0.02865 | 0.03878 |
| His | 4.9792 | 0.030897 | 0.041592 |
| lysoPC a C28:1 | 4.9012 | 0.032125 | 0.042908 |
| DG(18:0_20:0) | 4.895 | 0.032225 | 0.042908 |
| Phe | 4.8303 | 0.033294 | 0.044092 |
| HArg | 4.8052 | 0.033722 | 0.044418 |

**Supplementary Table S1:** Significant features in the CTCL cells treated with Glabridin, and ERK inhibitor alone and in combination (One-way ANOVA followed by the Fisher's Least Significant Difference (LSD) test).
