# Supplementary material for: Metabolomics analyses reveal the crucial role of ERK in regulating metabolic pathways associated with the proliferation of human cutaneous T‐cell lymphoma cells treated with Glabridin
Source: Cell Prolif. 2024 Jun 30;57(9):e13701. doi: 10.1111/cpr.13701 (PMC11503255; doi:10.1111/cpr.13701)
Supplement: Supplementary file 9 — Supplementary Table S2. Metabolome view of the important metabolic pathways based on the significant features identified in Glabridin treated CTCL cells (H9) using the MetaboAnalyst 6.0 (https://www.metaboanalyst.ca/). The table displays eight columns including the metabolic pathway, match status, the p‐value, the −log10 (p) value, the Holm p‐value, false discovery rate (FDR) and the impact value. [file CPR-57-e13701-s010.docx]

| **Pathway** | **Total Cmpd** | **Hits** | **Raw p** | **-LOG10(p)** | **Holm adjust** | **FDR** | **Impact** |
| --- | --- | --- | --- | --- | --- | --- | --- |
| Thiamine metabolism | 7 | 1 | 9.41E-10 | 9.0264 | 3.86E-08 | 3.86E-08 | 0 |
| Taurine and hypotaurine metabolism | 8 | 2 | 8.74E-09 | 8.0584 | 3.50E-07 | 1.79E-07 | 0.42857 |
| Glycine, serine and threonine metabolism | 33 | 6 | 1.13E-07 | 6.9475 | 4.40E-06 | 1.54E-06 | 0.52589 |
| Glutathione metabolism | 28 | 5 | 1.93E-07 | 6.7154 | 7.32E-06 | 1.97E-06 | 0.11182 |
| Cysteine and methionine metabolism | 33 | 5 | 1.08E-06 | 5.9646 | 4.01E-05 | 8.37E-06 | 0.3604 |
| Pantothenate and CoA biosynthesis | 20 | 4 | 1.22E-06 | 5.912 | 4.41E-05 | 8.37E-06 | 0.04762 |
| Primary bile acid biosynthesis | 46 | 3 | 1.98E-06 | 5.7032 | 6.93E-05 | 1.16E-05 | 0.02493 |
| Tyrosine metabolism | 42 | 2 | 4.37E-06 | 5.3598 | 0.000148 | 2.24E-05 | 0.25057 |
| Purine metabolism | 70 | 3 | 1.33E-05 | 4.8759 | 0.000439 | 5.64E-05 | 0.02769 |
| Lysine degradation | 30 | 1 | 1.38E-05 | 4.8613 | 0.00044 | 5.64E-05 | 0 |
| Arginine and proline metabolism | 36 | 6 | 2.82E-05 | 4.55 | 0.000874 | 0.000102 | 0.24999 |
| Arachidonic acid metabolism | 44 | 1 | 2.98E-05 | 4.5259 | 0.000894 | 0.000102 | 0.27659 |
| Butanoate metabolism | 15 | 3 | 3.34E-05 | 4.4768 | 0.000967 | 0.000103 | 0.03175 |
| Glycerophospholipid metabolism | 36 | 2 | 3.53E-05 | 4.4527 | 0.000987 | 0.000103 | 0.04318 |
| Sphingolipid metabolism | 32 | 2 | 5.13E-05 | 4.2897 | 0.001386 | 0.00014 | 0.21576 |
| Biosynthesis of unsaturated fatty acids | 36 | 2 | 7.89E-05 | 4.1032 | 0.00205 | 0.000202 | 0 |
| Galactose metabolism | 27 | 2 | 0.000109 | 3.9629 | 0.002723 | 0.00025 | 0.14531 |
| Citrate cycle (TCA cycle) | 20 | 1 | 0.00011 | 3.9598 | 0.002723 | 0.00025 | 0.03273 |
| Starch and sucrose metabolism | 18 | 1 | 0.000127 | 3.8957 | 0.002924 | 0.000261 | 0.4207 |
| Neomycin, kanamycin and gentamicin biosynthesis | 2 | 1 | 0.000127 | 3.8957 | 0.002924 | 0.000261 | 0 |
| Arginine biosynthesis | 14 | 5 | 0.000143 | 3.8454 | 0.002998 | 0.000279 | 0.40609 |
| beta-Alanine metabolism | 21 | 4 | 0.000163 | 3.7883 | 0.003256 | 0.000291 | 0.45522 |
| Alanine, aspartate and glutamate metabolism | 28 | 6 | 0.000163 | 3.7869 | 0.003256 | 0.000291 | 0.621 |
| Porphyrin metabolism | 31 | 2 | 0.000175 | 3.7561 | 0.003256 | 0.0003 | 0 |
| Glyoxylate and dicarboxylate metabolism | 32 | 4 | 0.00019 | 3.7217 | 0.003256 | 0.000311 | 0.14815 |
| Tryptophan metabolism | 41 | 2 | 0.000213 | 3.6709 | 0.003414 | 0.000336 | 0.24798 |
| Histidine metabolism | 16 | 4 | 0.000311 | 3.507 | 0.004668 | 0.000473 | 0.22131 |
| Lipoic acid metabolism | 28 | 1 | 0.000332 | 3.4791 | 0.004668 | 0.000486 | 0.0017 |
| Propanoate metabolism | 22 | 2 | 0.000422 | 3.3749 | 0.005483 | 0.000596 | 0 |
| Nitrogen metabolism | 6 | 2 | 0.000455 | 3.3419 | 0.005483 | 0.000622 | 0 |
| Steroid hormone biosynthesis | 87 | 2 | 0.00049 | 3.3095 | 0.005483 | 0.000649 | 0.0356 |
| Pyrimidine metabolism | 39 | 2 | 0.000555 | 3.2556 | 0.005551 | 0.000711 | 0 |
| Nicotinate and nicotinamide metabolism | 15 | 1 | 0.001437 | 2.8424 | 0.012937 | 0.001786 | 0 |
| D-Amino acid metabolism | 15 | 1 | 0.067762 | 1.169 | 0.5421 | 0.081714 | 0 |
| Ubiquinone and other terpenoid-quinone biosynthesis | 18 | 1 | 0.13053 | 0.88429 | 0.91372 | 0.15291 | 0 |
| Selenocompound metabolism | 20 | 1 | 0.18245 | 0.73886 | 1 | 0.20779 | 0 |
| Valine, leucine and isoleucine biosynthesis | 8 | 3 | 0.39222 | 0.40647 | 1 | 0.43463 | 0 |
| Phenylalanine, tyrosine and tryptophan biosynthesis | 4 | 2 | 0.5906 | 0.2287 | 1 | 0.62089 | 1 |
| Phenylalanine metabolism | 8 | 2 | 0.5906 | 0.2287 | 1 | 0.62089 | 0.35714 |
| Valine, leucine and isoleucine degradation | 40 | 2 | 0.86232 | 0.064332 | 1 | 0.88388 | 0 |
| Steroid biosynthesis | 41 | 1 | 0.95108 | 0.021784 | 1 | 0.95108 | 0 |

**Supplementary Table S2:**
