# Supplementary material for: Metabolomics analyses reveal the crucial role of ERK in regulating metabolic pathways associated with the proliferation of human cutaneous T‐cell lymphoma cells treated with Glabridin
Source: Cell Prolif. 2024 Jun 30;57(9):e13701. doi: 10.1111/cpr.13701 (PMC11503255; doi:10.1111/cpr.13701)
Supplement: Supplementary file 10 — Supplementary Table S3. Metabolome view of the important metabolic pathways based on the significant features identified between Glabridin versus Glabridin + ERK inhibitor treated CTCL cells (H9) using the MetaboAnalyst 6.0 (https://www.metaboanalyst.ca/). The table displays eight columns including the metabolic pathway, match status, the p‐value, the −log10 (p)‐value, the Holm p‐value, false discovery rate (FDR) and the impact value. [file CPR-57-e13701-s011.docx]

| **Pathway Name** | **Total Cmpd** | **Hits** | **Raw p** | **-LOG10(p)** | **Holm adjust** | **FDR** | **Impact** |
| --- | --- | --- | --- | --- | --- | --- | --- |
| Thiamine metabolism | 7 | 1 | 9.66E-08 | 7.0149 | 3.96E-06 | 3.64E-06 | 0 |
| Glycine, serine and threonine metabolism | 33 | 6 | 1.78E-07 | 6.7507 | 7.10E-06 | 3.64E-06 | 0.52589 |
| Glutathione metabolism | 28 | 5 | 2.94E-07 | 6.531 | 1.15E-05 | 4.02E-06 | 0.11182 |
| Taurine and hypotaurine metabolism | 8 | 2 | 4.58E-07 | 6.3388 | 1.74E-05 | 4.70E-06 | 0.42857 |
| Cysteine and methionine metabolism | 33 | 5 | 1.04E-06 | 5.9813 | 3.86E-05 | 8.56E-06 | 0.3604 |
| Glyoxylate and dicarboxylate metabolism | 32 | 4 | 1.52E-06 | 5.8171 | 5.49E-05 | 1.04E-05 | 0.14815 |
| Primary bile acid biosynthesis | 46 | 3 | 3.79E-06 | 5.4216 | 0.000133 | 2.22E-05 | 0.02493 |
| Biosynthesis of unsaturated fatty acids | 36 | 2 | 8.99E-06 | 5.0463 | 0.000306 | 4.61E-05 | 0 |
| Arachidonic acid metabolism | 44 | 1 | 1.81E-05 | 4.7427 | 0.000597 | 8.24E-05 | 0.27659 |
| Pantothenate and CoA biosynthesis | 20 | 4 | 3.19E-05 | 4.4968 | 0.001019 | 0.000131 | 0.04762 |
| Purine metabolism | 70 | 3 | 4.34E-05 | 4.3627 | 0.001345 | 0.000162 | 0.02769 |
| Sphingolipid metabolism | 32 | 2 | 7.81E-05 | 4.1072 | 0.002344 | 0.000267 | 0.21576 |
| Lipoic acid metabolism | 28 | 1 | 0.000101 | 3.9959 | 0.002928 | 0.000318 | 0.0017 |
| Steroid hormone biosynthesis | 87 | 2 | 0.000155 | 3.8085 | 0.004352 | 0.000455 | 0.0356 |
| Arginine biosynthesis | 14 | 5 | 0.000447 | 3.3499 | 0.012064 | 0.001221 | 0.40609 |
| Galactose metabolism | 27 | 2 | 0.000522 | 3.2824 | 0.013569 | 0.00129 | 0.14531 |
| D-Amino acid metabolism | 15 | 1 | 0.000535 | 3.2717 | 0.013569 | 0.00129 | 0 |
| Arginine and proline metabolism | 36 | 6 | 0.00083 | 3.0809 | 0.01992 | 0.001891 | 0.24999 |
| Porphyrin metabolism | 31 | 2 | 0.000904 | 3.0439 | 0.02079 | 0.001951 | 0 |
| Butanoate metabolism | 15 | 3 | 0.001239 | 2.9069 | 0.027262 | 0.00254 | 0.03175 |
| Tyrosine metabolism | 42 | 2 | 0.001738 | 2.7599 | 0.036499 | 0.003393 | 0.25057 |
| Tryptophan metabolism | 41 | 2 | 0.002004 | 2.6982 | 0.040075 | 0.003734 | 0.24798 |
| Citrate cycle (TCA cycle) | 20 | 1 | 0.002305 | 2.6374 | 0.043792 | 0.004109 | 0.03273 |
| Alanine, aspartate and glutamate metabolism | 28 | 6 | 0.005003 | 2.3008 | 0.090058 | 0.008547 | 0.621 |
| Glycerophospholipid metabolism | 36 | 2 | 0.006374 | 2.1956 | 0.10836 | 0.010454 | 0.04318 |
| Histidine metabolism | 16 | 4 | 0.012396 | 1.9067 | 0.19833 | 0.019547 | 0.22131 |
| beta-Alanine metabolism | 21 | 4 | 0.015958 | 1.797 | 0.23937 | 0.024232 | 0.45522 |
| Valine, leucine and isoleucine biosynthesis | 8 | 3 | 0.018174 | 1.7405 | 0.25444 | 0.026612 | 0 |
| Propanoate metabolism | 22 | 2 | 0.019374 | 1.7128 | 0.25444 | 0.027391 | 0 |
| Valine, leucine and isoleucine degradation | 40 | 2 | 0.020453 | 1.6892 | 0.25444 | 0.027953 | 0 |
| Pyrimidine metabolism | 39 | 2 | 0.021213 | 1.6734 | 0.25444 | 0.028056 | 0 |
| Phenylalanine, tyrosine and tryptophan biosynthesis | 4 | 2 | 0.023454 | 1.6298 | 0.25444 | 0.029139 | 1 |
| Phenylalanine metabolism | 8 | 2 | 0.023454 | 1.6298 | 0.25444 | 0.029139 | 0.35714 |
| Ubiquinone and other terpenoid-quinone biosynthesis | 18 | 1 | 0.025497 | 1.5935 | 0.25444 | 0.030249 | 0 |
| Nitrogen metabolism | 6 | 2 | 0.025823 | 1.588 | 0.25444 | 0.030249 | 0 |
| Nicotinate and nicotinamide metabolism | 15 | 1 | 0.032765 | 1.4846 | 0.25444 | 0.037315 | 0 |
| Lysine degradation | 30 | 1 | 0.039661 | 1.4016 | 0.25444 | 0.043948 | 0 |
| Starch and sucrose metabolism | 18 | 1 | 0.249 | 0.60381 | 0.99598 | 0.26177 | 0.4207 |
| Neomycin, kanamycin and gentamicin biosynthesis | 2 | 1 | 0.249 | 0.60381 | 0.99598 | 0.26177 | 0 |
| Selenocompound metabolism | 20 | 1 | 0.31022 | 0.50834 | 0.99598 | 0.31797 | 0 |
| Steroid biosynthesis | 41 | 1 | 0.55831 | 0.25313 | 0.99598 | 0.55831 | 0 |

**Supplementary Table S3**
