# Supplementary material for: Metabolomics analyses reveal the crucial role of ERK in regulating metabolic pathways associated with the proliferation of human cutaneous T‐cell lymphoma cells treated with Glabridin
Source: Cell Prolif. 2024 Jun 30;57(9):e13701. doi: 10.1111/cpr.13701 (PMC11503255; doi:10.1111/cpr.13701)
Supplement: Supplementary file 11 — Supplementary Table S4. List of antibodies used. [file CPR-57-e13701-s005.docx]

| **ANTIBODY** | **MANUFACTURER** | **CATALOGUE NO.** |
| --- | --- | --- |
| PH2AX | SANTA CRUZ BIOTECHNOLOGY | SC-517348 |
| B-ACTIN | CELL SIGNALING TECHNOLOGY | 4967 |
| HMGB1 | CELL SIGNALING TECHNOLOGY | 3935 |
| GAPDH | SANTA CRUZ BIOTECHNOLOGY | SC47724 |
| CLEAVED CASPASE 3 | CELL SIGNALING TECHNOLOGY | 9661 |
| PARP | CELL SIGNALING TECHNOLOGY | 9542 |
| LC3 A/B | CELL SIGNALING TECHNOLOGY | 4108 |
| BECLIN1 | CELL SIGNALING TECHNOLOGY | 3738 |
| HSP60 | SANTA CRUZ BIOTECHNOLOGY | SC13115 |
| P-ERK 1/2 | CELL SIGNALING TECHNOLOGY | 4370 |
| ERK 1/2 | CELL SIGNALING TECHNOLOGY | 4695 |
| PP38 | CELL SIGNALING TECHNOLOGY | 9211 |
| P38 | CELL SIGNALING TECHNOLOGY | 9212 |
| PJNK | CELL SIGNALING TECHNOLOGY | 4668 |
| JNK | CELL SIGNALING | 9252 |
| PAMPK A | CELL SIGNALING TECHNOLOGY | 2535 |
| AMPK A | CELL SIGNALING TECHNOLOGY | 2532 |
| C-MYC | CELL SIGNALING TECHNOLOGY | 9402 |
| NOTCH 1 | CELL SIGNALING TECHNOLOGY | 3608S |
| PAKT(S473) | CELL SIGNALING TECHNOLOGY | 4060 |
| AKT | CELL SIGNALING TECHNOLOGY | 9272 |
| A-TUBULIN | CELL SIGNALING TECHNOLOGY | 2144 |
| CLEAVED CASPASE 8 | CELL SIGNALING TECHNOLOGY | 9496 |
| CASPASE 3 | CELL SIGNALING TECHNOLOGY | 9662 |

**Supplementary Table S4:** List of antibodies used.
